# Variations in the breeding behavior of cichlids and the evolution of the multi-functional seminal plasma protein, seminal plasma glycoprotein 120

**DOI:** 10.1186/s12862-018-1292-0

**Published:** 2018-12-20

**Authors:** Masaya Morita, Stanley Ifeanyi Ugwu, Masanori Kohda

**Affiliations:** 1 Sesoko Station, Tropical Biosphere Research Center, University of the Ryukyus, Sesoko, Motobu, Okinawa, 905-0227 Japan; 2 Laboratory of Animal Sociology, Department of Biology and Geosciences, Graduate School of Sciences, Osaka City University, Sumiyoshi, Osaka, 558–8585 Japan

**Keywords:** Seminal plasma protein 120, Glycosylation, Cichlids, Positive selection, Correlated evolution, Fertilization success, Sperm competition

## Abstract

**Background:**

Seminal plasma proteins are associated with successful fertilization. However, their evolutionary correlation with fertilization mechanisms remains unclear. Cichlids from Lake Tanganyika show a variety-rich spawning behavior that is associated with the transfer of the sperm to the egg for fertilization. One of these behaviors, called “oral fertilization,” emerged during their speciation. In oral fertilization, females nuzzle the milt from male genitalia and pick up the released eggs in their mouths, which are then fertilized inside the oral cavity. Thus, the success of the fertilization is dependent on the retention of sperm in the oral cavity during spawning. Sperm aggregation and immobilization in viscous seminal plasma may help retain the sperm inside the oral cavity, which ultimately determines the success of the fertilization. Seminal plasma glycoprotein 120 (SPP120) is one of the major seminal plasma proteins present in cichlids. SPP120 has been implicated to immobilize sperm and increase the milt viscosity. However, the functional linkage between oral fertilization and seminal plasma proteins has not been investigated.

**Results:**

During trials of simulated oral fertilization, it was observed that milt viscosity contributed to fertilization success by facilitating longer retention of the milt inside the mouth during spawning. Glycosylation of SPP120 was associated with high milt viscosity. Its glycosylation was specifically present in the milt of cichlid species exhibiting oral fertilization. Moreover, recombinant SPP120 from several the oral fertilization species strongly immobilized/aggregated sperm. Therefore, the functions of SPP120 (immobilization/aggregation and its glycosylation) may contribute to success of oral fertilization, and these functions of SPP120 are more prominent in oral fertilization species. In addition, comparative phylogenetic analyses showed a positive evolutionary correlation between SPP120 function and oral fertilization. Hence, these evolutions may have occurred to keep up with the transition in the mode of fertilization. In addition, rapid evolution in the molecular sequence might be associated with functional modifications of SPP120.

**Conclusion:**

These results suggest that SPP120 might be associated with oral fertilization. In other words, reproductive traits that define the interaction between sperms and eggs could be the evolutionary selective force that cause the rapid functional modification of the fertilization-related reproductive protein, SPP120.

**Electronic supplementary material:**

The online version of this article (10.1186/s12862-018-1292-0) contains supplementary material, which is available to authorized users.

## Introduction

Reproductive traits (spawning behavior, sperm competition, and cryptic female choice) are closely associated with the role of seminal plasma proteins. Indeed, these traits, such as sperm competition, influence the molecular evolution of seminal plasma proteins [1–5]. Although the association between the molecular evolution of these proteins and reproductive traits has been explored (e.g., seminal plasma proteins form plugs to prevent the entry of another male’s semen in polyandrous species), studies on the functional modification of seminal plasma proteins due to molecular evolution (e.g., positive selection), post-translational modifications (e.g., glycosylation), and mRNA expression associated with the changes in the reproductive behavior are limited [6, 7]. For example, it has rarely been shown that changes in plug formation, ultimately leading to changes in the mating system, are a consequence of positive selection of specific codon mutations in seminal plasma proteins.

**Fig. 1:**
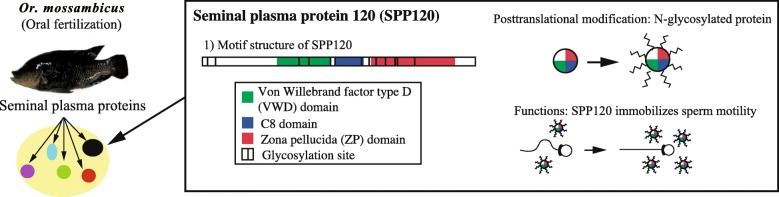
Motif structure, glycosylation, and function of seminal plasma protein 120 (SPP120). Seminal plasma protein 120 (SPP120) has many glycosylation sites, c8 domain, vWD domain, and ZP domain. One of function of SPP120 is immobilization of sperm motility [9]

SPP120 is a species-specific glycoprotein found only in cichlids [6, 8]. It is composed of three domains (von Willebrand factor type D, C8, and zona pellucida) containing several glycosylation sites (Fig. 1) [8]. These domains are thought to be involved in spermatozoa immobilization via adhesion of sperm with SPP120 and polymerization of SPP120 [8]. As a major glycoprotein in the seminal plasma of the cichlid *Oreochromis niloticus* [8, 9]*.* SPP120 may immobilize/aggregate sperm and may be related to the formation of colloidal and highly viscous milt. In support of this hypothesis, it has been observed that sperm motility is suppressed by SPP120 [9] and milt becomes colloidal [10], which may be due to SPP120 glycosylation. Further, SPP120 is a rapidly evolving gene that appears to have evolved through the process of gene conversion [6]. However, it is not known whether SPP120 regulates sperm motility and if the gene encoding this protein evolves rapidly.

The adaptive evolution of SPP120 as a sperm motility regulator may be related to the interaction between the sperm and egg from ejaculation to fertilization, which majorly depends on reproductive behaviors (i.e., timing of sperm ejaculation, number of ejaculating males trying for successful fertilization with limited number of eggs). For example, cichlids in Lake Tanganyika show distinctive spawning behaviors that define distinctive fertilization types (see below) [11–15]. In addition, sperm collection by females during spawning may cause sperm competition and cryptic female choice before fertilization [13, 16]. Therefore, complex behavioral traits must be examined.

**Fig. 2:**
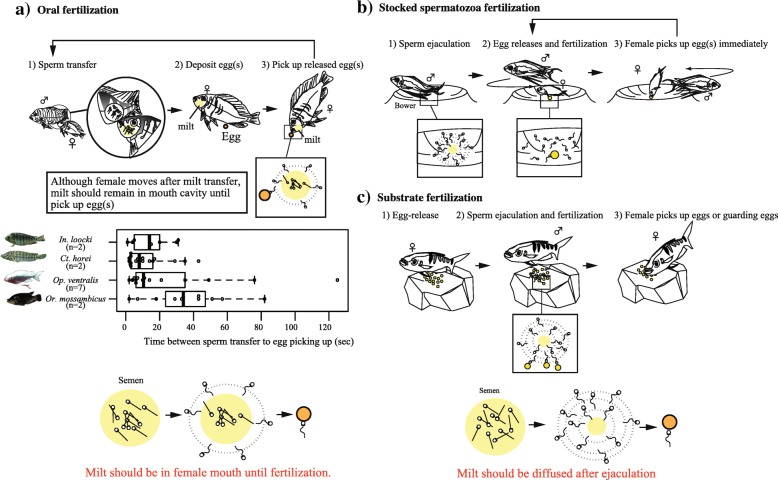
Fertilization types in cichlids inhabiting Lake Tanganyika. Spawning behavior in three distinct fertilization types: a) oral fertilization, b) stocked-spermatozoa fertilization, and c) substrate fertilization. In oral fertilization species (a), 1) female nuzzles milt from male genitalia (SI movie 1) or via pelvic fins (SI movie 2), and then 2) female deposits eggs and 3) picks up egg(s). Finally fertilization occurs in the mouth with the milt remaining in the mouth (diagram in a-3). In oral fertilization (a), the time between egg-release and sperm nuzzling is shown for *Interochromis loocki* (*N* = 2), *Ctenochromis horei* (N = 2), *Ophthalmotilapia ventralis* (*N* = 7), and *Oreochromis mossambicus* (N = 2). In oral fertilization species, milt should be in the mouth after milt transfer to female mouth until female mouth. In stocked spermatozoa fertilization (b), 1) sperm were released on the spawning sites called “bower” (diagram b-1), and female enters the bower. Female chases lekking male, 2) deposits eggs and 3) picks it up. Time between egg-release and picking up is short [15] and female turns position immediately to chase the male, female may not collect milt directly from the male (SI movie 3). Milt should diffuse immediately in the bower (diagram b-1) and fertilization may occur with pre-released sperm in the bower (diagram b-2). In substrate fertilization (c), 1) female deposits eggs on the ground or inside of the snail and 2) male comes to ejaculate sperm. Fertilization may occur after ejaculation (diagram c-2). Milt characteristics contributing to the success of each fertilization type are also shown

Among the different behavioral traits, spawning behavior may affect SPP120 evolution. A unique spawning behavior called “oral fertilization” is associated with the functions of SPP120. Presumably, SPP120 functions include sperm immobilization/aggregation and viscous milt formation, which might facilitate oral fertilization. Oral fertilization occurs in an enclosed space, i.e., the oral cavity (Fig. 2a; Additional files 1 and 2: Movies S1 and S2) [16–18]. In this spawning behavior, males deposit their milt into the female’s mouth before, during, and after egg release (Fig. 2a; Additional files 1 and 2: Movies 1 and 2) [13, 15]. When spawning occurs, the fertilization rate is supposed to increase if the available sperms remain in the oral cavity. Therefore, it is hypothesized that SPP120 plays a role in oral fertilization. However, many other cichlid species show “non-oral fertilization” spawning behaviors [e.g., 11, 12, 15], and thus, categorization of the fertilization types based on spawning behaviors is necessary.

Reproductive tactics, such as sneaking and cryptic female-choice, also possibly influence milt characteristics because of SPP120 evolution. In species that show oral fertilization, it has been reported that females collect milt from many males and these species also exhibit sperm competition and cryptic female-choice [13, 16]. In these species, the sperm must remain long enough in the mouth of the female to compete with the sperm from other males. As mentioned above, viscous milt formation and sperm immobilization/aggregation by SPP120 may contribute to fertilization success. However, in species that do not show oral fertilization, sneaking male release sperms prior to the territorial male [19–21]. The effect of sperm competition and cryptic female-choice should be examined along with fertilization types.

Since cichlids in the Lake Tanganyika speciated over a relatively short time span, and their reproductive traits, such as spawning behaviors, appear to have diversified rapidly, the functions of SPP120 (Fig. 1: spermatozoa aggregation/immobilization and milt viscosity) may also have changed according to the reproductive traits (spawning behaviors and/or reproductive tactics). Along with the proposed rapid diversification of reproductive traits, the varying functions of SPP120 would have arisen rapidly; it has also been reported that SPP120 has evolved rapidly [6]. However, despite the recognized importance of reproductive behavior in cichlid ecology and evolution [22–24], the relationship between reproductive traits and SPP120 function has not yet been explored.

Although the influence of reproductive behaviors on the evolution of the function of SPP120 has been implicated, the following questions remain unanswered: 1) Which fertilization types or reproductive tactics affected SPP120 evolution? 2) If fertilization types had affected SPP120 evolution, was its evolution associated only with oral fertilization? 3) Which functions of SPP120 were influenced by reproductive behaviors? 4) Has SPP120 evolution caught up with the diversification of cichlid reproductive behaviors in Lake Tanganyika? To answer these questions, integrative approaches involving biochemical studies, comparative phylogenetic analyses, and molecular sequence analyses were performed.

## Results

### Classification of fertilization types based on spawning behaviors

In this study, based on the spawning behavior of cichlids in Lake Tanganyika, we classified the type of fertilization into three categories, namely, fertilization in the oral cavity (oral fertilization; Fig. 2a; Additional files 1 and 2: Movies S1 and S2), fertilization using pre-released spermatozoa in a “bower” (stocked-spermatozoa fertilization; Fig. 2b; Additional file 3: Movie S3), and substrate fertilization in substrate brooders or mouth brooders (Fig. 2c; Additional file 4: Movie S4).

In oral fertilization cascades, males deposit their milt into the female’s mouth before, during, and after egg release (Fig. 2a; Additional files 1 and 2: Movies S1 and S2) [13, 15]. Females approach egg-spots and egg-dummies to collect milt. The time between nuzzling milt to egg release varied from 2 s to more than 2 min (Fig. 2a) [15]. *Ophthalmotilapia ventralis* and *Ctenochromis horei* females spawned only one to two eggs at a time and then picked them up.

In substrate- or stocked-spermatozoa fertilization, spermatozoa are released at spawning sites or onto the released eggs (Fig. 2b and c; Additional files 3 and 4: Movies S3 and S4) [11, 21, 25, 26]. In stocked-spermatozoa fertilization, the male builds a crater-shaped spawning site known as a “bower” (Fig. 2b; Additional files 3: Movie S3) [27], releases its spermatozoa [15], and the female then spawns into the bottom of the bower. Since the female has to pick up the egg(s) immediately from the bower, the female chases the lekking male to collect its milt in this limited time (Fig. 2b; Additional file 3: Movie S3) [14, 28]. Fertilization may occur via pre-released spermatozoa (Fig. 2b; Additional file 3: Movie S3) [15]. In substrate fertilization, which includes mouth and substrate brooders, females deposit their eggs before ejaculation (Fig. 2c; Additional file 4: Movie S4) [11, 21, 25, 26].

### Oral fertilization might have arisen during the speciation of Tanganyikan cichlids

**Fig. 3:**
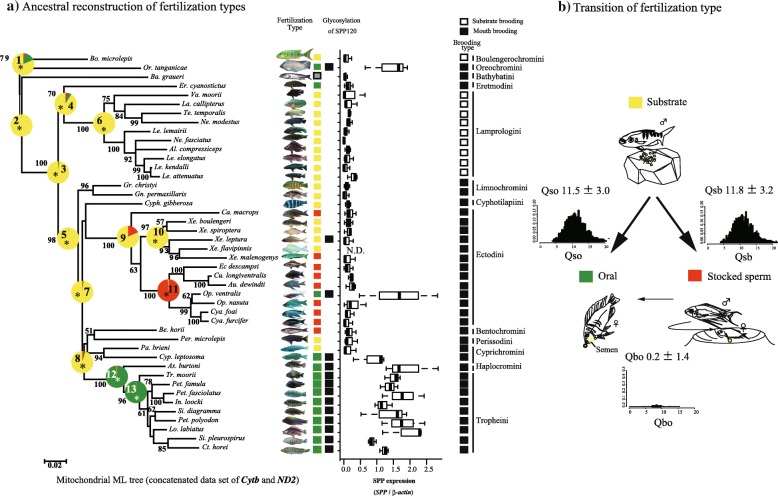
Transitions of fertilization types during speciation. Transitions and ancestral reconstruction of fertilization types for 44 cichlids from Lake Tanganyika were shown. **a)** Ancestral states of nodes (1–13) were shown and statistical tests were carried out in terms of likelihood ratios (2ΔlnL) (Additional file 7: Table S2). Asterisks indicate that ancestral states of the fertilization are statistically dominant (Additional file 7: Table S2). Fertilization types, the glycosylation state of SPP120, and the expression of *SPP120* in relation to *β-actin* (*N* = 3 each species) are also shown. **b)** Transition rates and ancestral states were calculated in BayesTraits version 2.0. Arrows indicate transition rates (Oso, Qsb, Qbo), which are an indicator of how likely a particular transition is to have occurred. Qso indicates rates from substrate fertilization to oral fertilization, Qsb is from substrate to stocked sperm fertilization, and Qbo is from stocked-sperm fertilization to oral fertilization. Histograms of each transition rate are also shown

Ancestral reconstruction with both mitochondrial and nuclear markers showed that substrate fertilization dominated the nodes of deep lineages (node 1 to 10, except node 9) and the other two fertilization types dominated the group that speciated later (Fig. 3a; Additional files 5 and 6: Figure S1 and Table S5; Additional file 7: Table S2), suggesting that oral fertilization and stocked-spermatozoa fertilization arose during speciation. In addition, analyses with BayesTraits version 2.0 [29, 30] indicated that transitions from substrate fertilization to the other fertilization types occurred frequently (Fig. 3b; Additional file 8: Figure S2) and that the transition rates from substrate fertilization to the other fertilization types (Qsb and Qso) were high (Fig. 3b). The comparison of their harmonic means verified that transitions from substrate fertilization to other fertilization types were highly probable in the mitochondrial tree (Bayes Factor = 13.48; Additional file 8: Figure S2), but these were not so strongly supported in the nuclear tree (Bayes Factor = 1.0). However, such differences were due to the use of different species in each tree. For example, the nuclear marker tree did not include *Op. ventralis* and *Ophthalmotilapia nasuta* that changed from stocked-spermatozoa to oral fertilization.

### Viscous milt contributes to success of oral fertilization

**Fig. 4:**
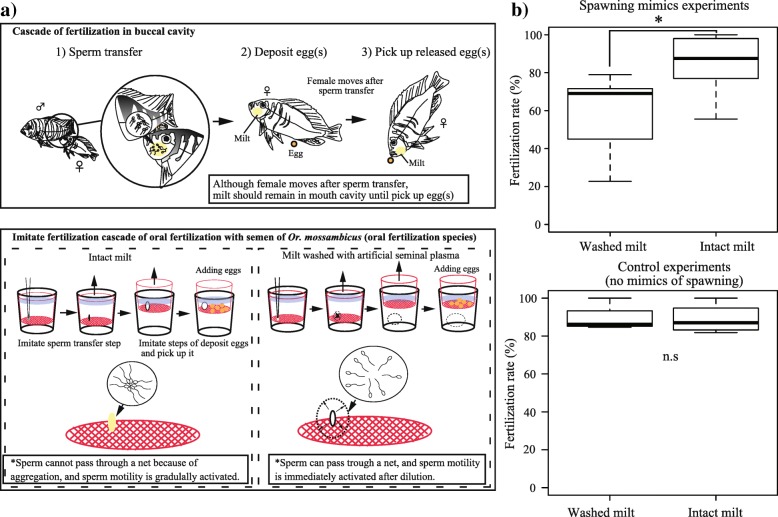
Fertilization trials in *Or. mossambicus* to imitate oral fertilization cascades with viscous milt or non-viscous milt. **a** Schema of fertilization trials with intact viscous milt or washed diffusible milt. **b** Fertilization rates with washed or intact milt (N = 7, 20 to 30 eggs were used for each trials)

To investigate if seminal plasma contributed to oral fertilization success, trials simulating the oral fertilization cascades were performed in the Mozambique tilapia *Oreochromis mossambicus,* using intact viscous milt or milt washed with artificial seminal plasma (ASP) lacking SPP120 (Fig. 4a). The fertilization success was lower among ASP-washed milt than that among intact milt samples (washed milt: 51.2% ± 7.8%; intact milt: 84.2% ± 7.9%; paired t-test *P* < 0.005, *t* = − 4.64, d.f.. = 6; Fig. 4b). However, in fertilization trials without the simulation of the oral fertilization cascade, fertilization rates using ASP washed sperm were not significantly different from that of intact sperm (Fig. 4b: *P* > 0.05, *t* = − 0.04, d.f. = 3). Thus, viscous seminal plasma is related to oral fertilization success.

### Glycosylated SPP120 is associated with milt viscosity

**Fig. 5:**
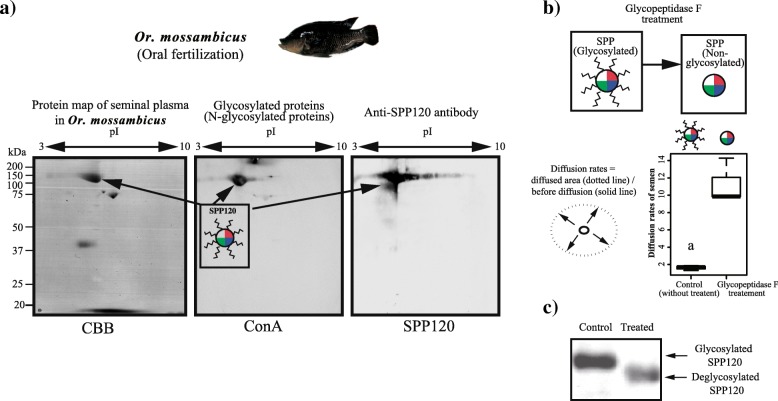
Glycosylation of SPP120 is major protein in seminal plasma and involved in milt viscosity in the oral fertilization species *Oreochromis mossambicus* (**a**) Maps of seminal plasma proteins (Coomassie brilliant blue staining), N-glycosylated proteins (ConA lecting blotting), and Anti-SPP120 antibody reacted protein in the oral fertilization species *Oreochromis mossambicus*. Glycopeptidase-F treatment was applied to seminal plasma, and its effect on **b** milt viscosity and **c** the glycosylation states of SPP120 and was observed. Milt diffusion rates were calculated as the ratio of the diffused area just after glycopeptidase-F inoculation and 3 s later (N = 3) and compared between control and peptidase-treated milt (*t*-test d.f. = 2.05, *t* = 6.52, *P* < 0.02)

The correlation between milt viscosity and seminal plasma glycoproteins was analyzed in the species *Or. Mossambicus*, which shows oral fertilization, via 2-D electrophoresis, lectin blotting using Concanavalin A (ConA), and by using an anti-SPP120 antibody. The protein map of the seminal plasma showed that the largest protein spot, SPP120, was N-glycosylated, whereas the other proteins were not strongly glycosylated (Fig. 5a).

To check if SPP120 glycosylation is associated with milt viscosity, glycopeptidase F, which removes the glycol chain from proteins, was applied to the milt of *Or. mossambicus*. After treatment with glycopeptidase F, milt viscosity decreased (Fig. 5b; T-test *P* < 0.02, *t* = 6.52), as well as the molecular mass of the positive control (Fig. 5c). Upon glycosylation, the molecular mass of SPP120 increases from approximately 90 kDa (non-glycosylated) to 120 kDa (glycosylated) [8, 9]. Thus, the results suggest that SPP120 is a major glycoprotein contributing to the highly viscous milt observed in *Or. mossambicus*.

### SPP120 glycosylation is evolutionarily correlated with oral fertilization

**Fig. 6:**
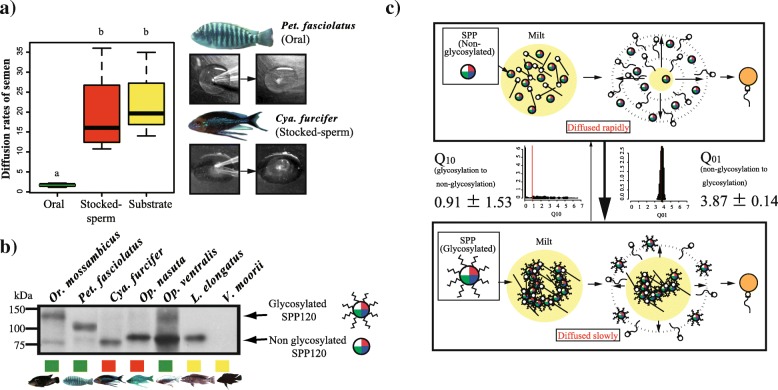
Differences in milt diffusion rates and glycosylation states of SPP120 among the fertilization types of cichlids from Lake Tanganyika. **a** Milt diffusion was evaluated in seven species (see also Additional file 11: Figure S4) and compared (Phylogenetic generalized least squares *P* < 0.005; see also Additional file 10: Table S3). Oral fertilization species: *Or. mossambicus* (N = 3), *Petrochromis fasciolatus* (*N* = 4), and *Ophthalmotilapia ventralis* (N = 4); Stocked-spermatozoa fertilization species: *Cyathopharynx furcifer* (N = 3) and *Op. nasuta* (N = 3); and substrate fertilization species: *Variabilichromis moorii* (N = 3), and *Lepidiolamprologus elongatus* (N = 3). **b** Glycosylation states of SPP120 in these seven species are also shown. **c** Transitions among glycosylation states were calculated in BayesTraits version 2.0 according to maximum likelihood estimations. Arrows indicate transition rates (O_01_, Q_10_), which are an indicator of how likely a particular transition is to have occurred. Q_01_ indicates rates from non-glycosylated SPP120 to glycosylated one, Q_10_ is from glycosylated SPP120 to non-glycosylated one. Histograms of each transition rate are also shown

Milt viscosity and SPP120 glycosylation were examined in 7 and 44 species of Tanganyikan cichlids, respectively. Milt diffusion was significantly slower in species with oral fertilization than in species with other fertilization types. In the species with oral fertilization, i.e., *Petrochromis fasciolatus, Or. mossambicus,* and *Op. ventralis*, the milt diffused slowly because of its high viscosity (Fig. 6a; Additional file 9: Figure S3). In contrast, milt diffused rapidly in the species with stocked-spermatozoa fertilization, i.e., *Cyathopharynx furcifer* and *Op. nasuta*, as well as in the species with substrate fertilization, i.e., *Variabilichromis moorii* and *Lepidiolamprologus elongatus* (Fig. 6b; Additional file 7: Figure S3; Additional file 10: Table S3; Phylogenetic generalized least squares (PGLS) *P* < 0.001).

Among forty-four species, glycosylated SPP120 was detected mostly in the species with oral fertilization but not in the species with other fertilization types (Fig. 3a; Fig. 6b; Additional file 11: Figure S4; Additional file 12: Table S1). A typical glycosylation of SPP120 is shown for seven species in Fig. 6b. Further, SPP120 was not detected in several substrate fertilization species, including *Va. moorii* and *Boulengerochromis microlepis*.

To assess the evolutionary correlation between the species with oral fertilization and SPP120 glycosylation, PGLS analyses were performed using mitochondrial and nuclear marker trees. Although the representative phylogenetic signal, i.e., the maximum likelihood estimated lambda (λ), differed between trees (Mitochondrial tree λ = 0, Nuclear tree λ = 1; see methods section about lambda value), both trees showed a strong positive correlation between glycosylation and oral fertilization (Table 1). Intensity of sperm competition was weakly correlated with glycosylation in nuclear markers (Table 1). The difference between lambda values might be due to having different number of taxa in each tree (mitochondrial tree: 44, nuclear tree: 20). However, lambda value is not reliable for less than 20 taxa [31]. Moreover, SPP120 glycosylation and milt diffusion rates were negatively correlated (PGLS with mitochondrial tree: 4). Although the lambda values differed between trees, it was observed that there was a positive correlation between the SPP120 glycosylation causing viscous milt and oral fertilization.

**Table 1:** Results from phylogenetic generalized least squares (PGLS) analyses

Trait	R2	F	df	p	Lambda	Predictors	Coefficient(estimated)	Std. Error	t	P
Mitochondrial tree										
Glycosylation	0.74	30.11	4, 38	< 0.001	Estimated lambda: 1	Stocked-sperm fertilization	−0.18	0.19	−0.92	0.36
					lower bound: 0.000, *p* < 0.0001	Substrate fertilization	0.03	0.17	0.17	0.86
					upper bound: 1.000, *p* = 1	Oral fertilization	0.82	0.19	4.41	< 0.001*
						Sperm competition	0.01	0.027	0.50	0.62
*SPP* expression	0.70	21.8	4,38	< 0.001	Estimated lambda: 0.64	Stocked-sperm fertilization	−0.14	0.28	−0.52	0.61
					lower bound: 0.000, p = 1	Substrate fertilization	0.002	0.22	0.008	0.99
					upper bound: 1.000, *p* = 0.001	Oral fertilization	1.10	0.24	4.66	< 0.001*
						Sperm competition	0.01	0.027	0.50	0.62
Sperm immobilization by N-terminal region of SPP120	0.94	47.4	4,7	< 0.001	Estimated lambda: 1	Stocked-sperm fertilization	0.31	0.14	2.21	0.06
					lower bound: 0.000, *p* = 0.02	Substrate fertilization	0.19	0.08	2.49	0.04*
					upper bound: 1.000, p = 1	Oral fertilization	−0.3	0.07	−3.87	< 0.001*
						Sperm competition	−0.04	0.02	−1.72	0.12
Nuclear markers										
Glycosylation	0.84	21.7	4.16	< 0.001	Estimated lambda: 0	Stocked-sperm fertilization	−0.32	0.28	−1.16	0.27
					lower bound: 0.000, p = 1	Substrate fertilization	−0.16	0.21	−0.78	0.45
					upper bound: 1.000, *p* = 0.09	Oral fertilization	0.65	0.22	2.97	< 0.01*
						Sperm competition	0.11	0.05	2.22	0.04*
*SPP* expression	0.72	10.5	4,16	< 0.001	Estimated lambda: 0	Stocked-sperm fertilization	−0.13	0.53	−0.24	0.81
					lower bound: 0.000, p = 1	Substrate fertilization	−0.07	0.39	−0.18	0.86
					upper bound: 1.000, *p* = 0.01	Oral fertilization	1.03	0.41	2.49	0.02*
						Sperm competition	0.09	0.09	1.03	0.32
Sperm immobilization by N-terminal region of SPP120 *tree of SPP120 was used	0.89	24.9	4,7	< 0.001	Estimated lambda: 0	Stocked-sperm fertilization	0.39	0.09	4.08	< 0.005*
					lower bound: 0.000, p = 1	Substrate fertilization	0.22	0.06	3.73	< 0.001*
					upper bound: 1.000, *p* = 0.50	Oral fertilization	−0.24	0.06	−3.86	< 0.005*
						Sperm competition	−0.06	0.02	−2.58	0.04*

Transitions in glycosylation states also concurred with fertilization types. As mentioned above, oral and stocked-spermatozoa fertilization might have arisen from substrate fertilization (Fig. 3a and b). SPP120 glycosylation was specifically observed in oral fertilization and the transition from non-glycosylated to glycosylated SPP120 was highly probable (Fig. 6c). Discrete analysis showed that oral fertilization and glycosylation were strongly correlated (likelihood ratios between independent and dependent models: mitochondrial tree 2ΔLn = 18.55, *P* < 0.001; Nuclear tree 2ΔLn = 20.65, *P* < 0.001; Additional file 13: Figure S5).

Viscous milt, caused by SPP120 glycosylation, is deeply associated with oral fertilization success. However, because rapid diffusion and spermatozoa activation is required for successful fertilization in substrate- and stocked-spermatozoa fertilization [15, 32, 33], the immobilization of spermatozoa due to SPP120 is not likely to contribute to these types of fertilizations. Therefore, this raises the question of what the role of SPP120 might be in species that do not exhibit oral fertilization.

### SPP120 immobilizes and aggregates spermatozoa in species with oral fertilization

**Fig. 7:**
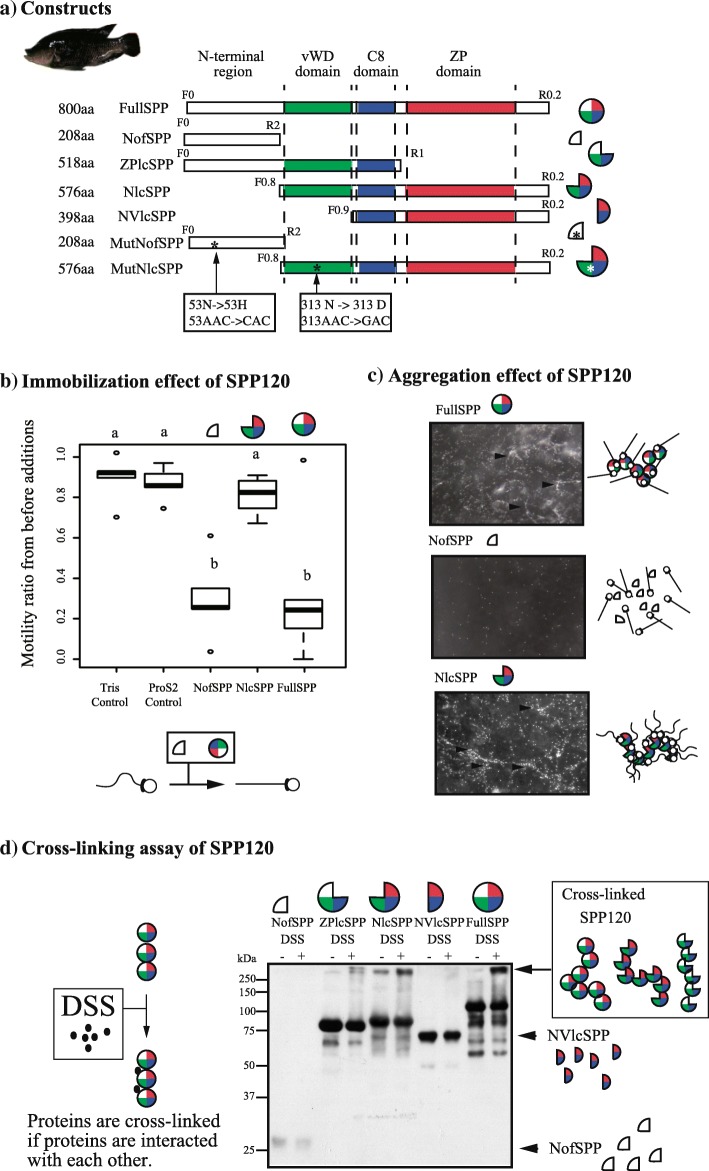
Functional differences in spermatozoa immobilization and aggregation by SPP120 (**a**) Seven SPP120 constructs were developed to investigate the function of each domain. (**b**) The effect of recombinant SPP120 on sperm motility was examined after the addition of expressed SPP (final concentration = 10 μg/ml). The percentage of mobile spermatozoa before and 30 s after the addition of SPP120 was calculated based on video recordings and statistically analyzed using Tukey’s HSD test (*N* = 5). (**c**) In the aggregation assay, spermatozoa were first suspended in 30 μl of a Ca^2+^-free activation solution containing expressed SPP120 (30 μg/ml); 3 μl of 100 mM CaCl_2_ were then added to observe sperm aggregation (N = 5). Arrowheads show the aggregated-sperm. (**d**) In the cross-linking assay, 10 μg of each protein were applied in SDS-PAGE after cross-linking with or without 100 μM DSS, and detected using anti-SPP120 antibody

To examine the effects of SPP120 on sperm motility, the functions of several types of recombinant SPP120 were examined (Fig. 7a). The addition of full length SPP120 (FullSPP) gradually immobilized spermatozoa (Fig. 7b; Additional file 14: Movie S5; Tukey HSD test, *P* < 0.05) and caused aggregation of spermatozoa (indicated by the arrowhead in Fig. 7c; Additional file 15: Movie S6). In contrast, the addition of an N-terminal region of SPP120 (NofSPP) suppressed sperm motility (Fig. 7b; Tukey HSD test, a vs. b, *P* < 0.005; Additional file 16: Movie S7) but did not aggregate spermatozoa (Fig. 7c). Adding SPP120 lacking N-terminal (NlcSPP) aggregated spermatozoa but showed continuous flagellar beating (Fig. 7b and c; Additional file 17: Movie S8; Tukey HSD test, *P* > 0.05). Additionally, ProS2 and tris buffer did not aggregate spermatozoa or inhibit their motility (Fig. 7b; Additional file 18: Movie S9; Tukey HSD test, *P* > 0.05). These results suggested that the N-terminal region of SPP120 contributes to sperm immobilization, and that the remaining domains are involved in sperm aggregation.

To evaluate the interaction of SPP120 with other SPP120 molecules and with sperm proteins, cross-linking experiments with disuccinimidyl suberate (DSS) and far-western analysis were performed. FullSPP, NlcSPP, and ZP-lacking SPP120 (ZPlcSPP) were cross-linked, whereas NofSPP and N-terminal and vWD domain lacking SPP120 (NVlcSPP) were not (Fig. 7d); ProS2 did not interact with itself (Additional file 19: Figure S6). These results suggest that the vWD domain is crucial to form SPP120 homo-polymers. As ZPlcSPP was cross-linked, the ZP domain was not functionally important to form homo-polymers. However, this domain might be involved in the interaction of SPP120 with spermatozoa. The far-western analysis using FullSPP showed that SPP120 interacted with the triton-soluble fraction of spermatozoa containing membrane proteins (Additional file 20: Figure S7), suggesting that SPP120 has affinity to sperm.

### Functional differences in SPP120 among species with distinct fertilization mechanisms

**Fig. 8:**
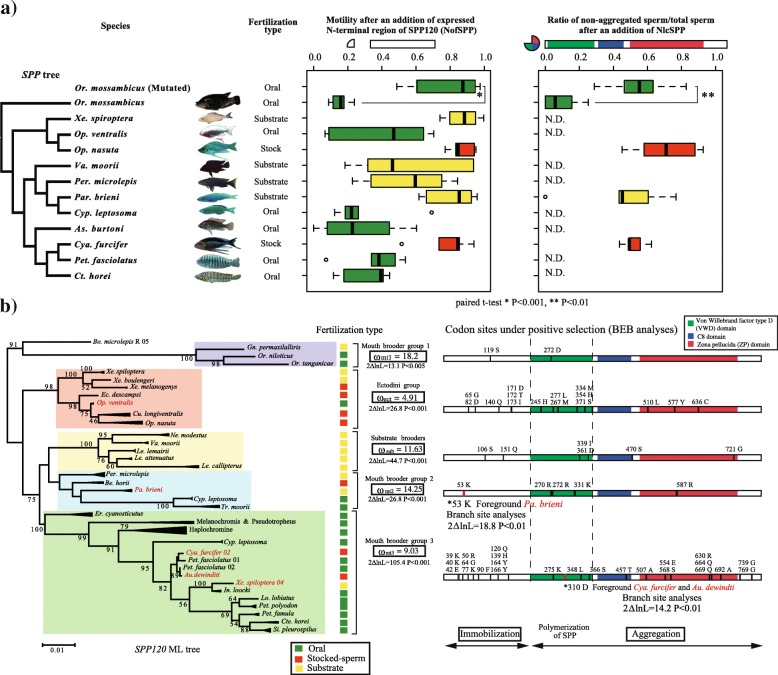
Functional differences of SPP120 according to fertilization types and molecular evolution of *SPP120* (**a**) The N-terminal region of SPP120 was checked in several species (*N* = 6 in NofSPP, N = 5 in NlcSPP). The ratio of sperm motility before and after the addition of proteins or Ca^2+^ (in percentage) is shown. Spermatozoa immobilization and aggregation were significantly stronger in oral fertilization than in the other types (NofSPP120: Phylogenetic generalized least squares *P* < 0.001, Additional file 22: Table S5; NlcSPP: Tukey’s HSD *P* < 0.05). (**b**) Maximum likelihood phylogenetic tree of *SPP120* and codon sites under positive selection within the five groups formed by *SPP120* copies (mouth brooder groups 1, 2, and 3, Ectodini, and substrate brooders). Functional *SPP120* copies among the five groups were applied in molecular evolutionary analyses (codon site model and branch-site analyses) regarding non-synonymous/synonymous mutations (ω) using CODEML in PAML [56]. Two models (null hypothesis: neutral and hypothesis: positive selection) were run for each analysis and their log-likelihoods were compared to assess if there was positive selection or not. Black bars indicate positively selected sites in codon site analyses and red bars indicate positively selected sites in branch site analyses. Species indicated in red were used as the foreground in branch site analyses to examine accelerated evolution

To examine functional differences of SPP120 according to fertilization types, expressed recombinant NofSPP or NlcSPP proteins from several species (NofSPP: twelve species, NlcSPP: four species) (Fig. 8a) were added to a sperm suspension of *Or. mossambicus*. The immobilization effects of NofSPP on substrate or stocked-spermatozoa species were weaker than that of species with oral fertilization (Fig. 8a; Additional file 21: Figure S8; Additional file 22: Table S5, oral v/s. substrate or stocked-spermatozoa, *P* < 0.001). Therefore, even though no species-specific immobilization effect was observed, the 12 species from different clades consistently presented a functional differentiation in SPP120 based on fertilization type (Fig. 8a). The evolutionary relationship between fertilization type and sperm motility following the addition of NofSPP was negatively correlated with oral fertilization (PGLS analyses with both mitochondrial and nuclear tree; Table 1), whereas, the other fertilization mechanisms did not show any correlation with sperm immobilization (Table 1). Therefore, oral fertilization may enhance the immobilization function of NofSPP as spermatozoa immobilization contributes to fertilization success. On the contrary, the immobilization function was weaker in the other fertilization types.

The aggregation effect was examined after the addition of the expressed NlcSPP proteins. The stocked-spermatozoa fertilization species *Cya. furcifer* and *Op. nasuta* did not exhibit sperm aggregation (Fig. 8a; Tukey HSD test, *P* < 0.005 in *O. mossambicus* vs. *Cya. furcifer* or *Op. nasuta*), and the substrate fertilization species *Paracyprichromis brieni* did not strongly aggregate *Or. mossambicus* spermatozoa (Fig. 8a; Tukey HSD test, *P* = 0.04). Although it was not clear whether this weakened aggregation was related to fertilization type or if it was species-specific, the aggregation effects in non-oral fertilization species were weaker than those in oral fertilization species (Table 1.).

Overall, the immobilization and aggregation functions of SPP120 resulted from its molecular evolution may have become suitable to each fertilization type. Analysis of molecular evolution was conducted in order to understand the evolution of functional differences developed in response to fertilization types.

### The molecular evolution of SPP120 was influenced by changes in fertilization types

To explore if differences in fertilization type affected the rate of molecular evolution of SPP120 in Tanganyikan cichlids, cDNA sequences of this gene were isolated from 31 species showing distinct fertilization types. These sequences were classified into five groups based on the Maximum likelihood (ML) tree (mouth brooder groups 1, 2, and 3, Ectodini group, and substrate brooder group; Fig. 8b; Additional file 23: Figure S9). Mouth brooder group 3 contained gene copies of several species belonging to the tribe Ectodini, although most species belonged to the tribes Tropheini and Haplochromini. These results may be the due to the incomplete lineage sorting in Tanganyikan cichlids during speciation [34, 35], and thus, several gene copies might belong to different groups. In mouth-brooding cichlids that undergo oral fertilization [36], many copies of SPP120 were conserved over all domains (Additional file 23: Figure S9; Additional file 24: Table S6). In contrast, many copies of SPP120 in group Ectodini and mouth brooder group 2 were pseudogenes (Additional file 23: Figure S9; Additional file 24: Table S6). Most pseudogenes were not present in species with oral fertilization, but many pseudogenes that were present in substrate brooders were functional copies (Fig. 8b; Additional file 23: Figure S9; Additional file 24: Table S6).

To evaluate the evolution of functional modifications in SPP120, two types of analysis were carried out to detect positive selection in five SPP120 groups: codon-site analysis in each group to detect selected sites along SPP120, and branch-site analysis to detect specific positively selected codon sites in response to changes in fertilization types.

The codon site model supported positive selection (codon site model; model 8 vs. 8a) in all SPP120 groups (Fig. 8b; Additional file 25: Table S7) and the Bayes empirical Bayes (BEB) analysis of each group revealed that specific sites were selected in each group, with the exception of substrate brooders (Fig. 8b; Additional file 25: Table S7). In mouth brooder 3 and Ectodini groups, the N-terminal region and vWD domain were positively selected (Fig. 8b; Additional file 25: Table S7). These sites are functionally important for spermatozoa immobilization and SPP120 polymerization (Fig. 7b, c, d), implying that these selections caused functional modifications in SPP120. Comparative phylogenetic analysis (PGLS) of NofSPP indicated that spermatozoa immobilization was negatively correlated with oral fertilization (Table 1). However, each SPP120 group had a distinct fertilization type (Fig. 8b; Additional file 23: Figure S9), and spermatozoa immobilization was weak in non-oral fertilization species in the functional assay (Fig. 8a). Thus, positive selection likely weakened or improved this function in each group.

To choose positively selected codon sites related to the functional modifications in SPP120, each branch representing a different fertilization type was set as the foreground within the group, and branch site analyses were performed (Fig. 8c; Additional file 26: Table S8). The branches of *Cya. furcifer* and *Aulonocranus dewindti* in mouth brooder group 3 and *Pa. brieni* in mouth brooder group 2 were significant (*P* < 0.01), while the branches of *Op. ventralis* in the Ectodini group were not (Fig. 8b; Additional file 26: Table S8). The BEB results indicated that two codon sites in the N-terminal region and vWD domain, which contributed to spermatozoa immobilization/aggregation, were under positive selection (Fig. 8b; Additional file 26: Table S8). Although NofSPP weakened the immobilization effect of SPP120 in *Cya. furcifer*, specific codon sites were not detected in the N-terminal region. Further, no specific positive selection sites were detected in the oral fertilization species *Op. ventralis*, which belongs to the Ectodini group that mostly comprises species with non-oral fertilization.

### Positive selection is related to functional modifications in SPP120

According to the functional assays and branch-site analysis, two codon sites were likely to be related to functional modifications in SPP120. To investigate this hypothesis, SPP120 point mutants were constructed to determine if positive selection influenced the functions of SPP120. The SPP120 mutation sites selected for the bower-building species *Cya. furcifer* (vWD domain) and *Pa. brieni* (N-terminal region) were introduced into the gene encoding SPP120 in the oral fertilization species *Or. mossambicus* (Fig. 7a and 8b). The mutated vWD domain of SPP120 (MutNlcSPP) did not induce spermatozoa aggregation to the same extent as that of intact NlcSPP (Fig. 8a; Paired t-test, *t* = − 5.444, d.f. = 4). Mutations in the N-terminal region of SPP120 (MutNofSPP) also weakened its immobilization function (Fig. 8a; Paired t-test, *P* < 0.01, *t* = − 7.6784, d.f. = 5). These data indicated that positive selection was related to functional modifications in SPP120.

### Differences in SPP120 expression according to fertilization types

The expression level of SPP120 examined using semi-quantitative PCR was significantly higher in species exhibiting oral fertilization than in those exhibiting substrate and stocked-spermatozoa fertilization (Fig. 3a; Additional file 27: Figure S10; Additional file 28: Table S9; PGLS, *P* < 0.001). The evolutionary correlation between fertilization type and expression level was positive in oral fertilization but not in the other fertilization types (Table 1). The lambda value of the analyses was 0.64 in the mitochondrial tree (44 species) and 0 in the nuclear tree (19 species), indicating that the correlation was slightly constrained by phylogeny. Intensity of sperm competition was not correlated.

## Discussion

We examined the evolution of SPP120 regarding its role in milt viscosity (SPP glycosylation) and sperm motility regulation in cichlids from Lake Tanganyika, which show distinctive fertilization types based on variations of spawning behaviors (substrate, stocked-spermatozoa, and oral fertilization) [11–15] that diverged during speciation. Our ancestral reconstructions suggest that oral fertilization and stocked-sperm fertilization arose in later speciated species (Fig. 3a, b). Since SPP120 contributes to the success of oral fertilization, the functions of SPP120 may have been influenced by the fertilization type to produce milt with traits that contribute the most to the success of each fertilization type. However, our results show that sperm competition might not have been closely related with SPP120 evolution. The processes occurring from ejaculation to fertilization are strongly influenced by SPP120 and thus, fertilization types are tightly associated with fertilization success with assistance of SPP120. In contrast, fertilization success might be partly affected by sperm competition, of which, level and intensity of sperm competition might be fluctuated among individuals.

Our results showed that SPP120 is involved in multiple aspects of milt viscosity and sperm motility regulation. The N-terminal region of SPP120 is likely to be involved in the immobilization of spermatozoa, whereas the remaining domains (vWD, C8, and ZP3) are possibly involved in aggregation (Fig. 7b, c). Further, the vWD domain is likely to be associated with SPP120 polymerization, which induces a stronger aggregation (Fig. 7c, d). These functionally important sites are under positive selection to cause functional modifications (Fig. 8b) and seem to be associated with milt traits as a large amount of SPP120 was detected in seminal plasma and its glycosylation resulted in viscous milt (Fig. 5; Fig. 6). The expression of *SPP120* differed according to fertilization types (Fig. 3a), and PGLS analysis showed that SPP120 functions were correlated with oral fertilization. However, the lambda values of maximum likelihood estimated for PGLS, which indicate the phylogenetic signal, differed between mitochondrial and nuclear tree (Table 1). The differences in lambda value might be because of species that treated in the trees, nuclear markers tree treated limited number of species. For example, several closely related species in mitochondrial tree that changed fertilization types are lacked (i.e., *Op. ventralis* and *Op. nasuta*).

In the species with oral fertilization (Fig. 2a; Additional files 1 and 2: Movie S1 and S2), functions of SPP120, milt viscosity, and spermatozoa immobilization/aggregation contributed to the success of oral fertilization. Given the spawning cascade (milt deposition in female mouth, release of egg(s), egg(s) collection by female, and fertilization in oral cavity; Fig. 2a; Additional files 1 and 2: Movie S1 and S2) observed in the species with oral fertilization, milt should remain in the female oral cavity and sperm motility should be gradually activated according to the slow diffusion of milt (Fig. 2a). Thus, spermatozoa immobilization and aggregation in viscous milt is likely a good tactic to prevent wastage of spermatozoa available for fertilization in the oral cavity. Success rate of oral fertilization in *Or. mossambicus* decreased when milt was washed with artificial seminal plasma (Fig. 4a, b). In addition, if sperm competition occurs in the female’s oral cavity because of sperm collection from many males, the sperm-retaining function of SPP120 is expected to significantly contribute in the success of fertilization. Therefore, both oral fertilization and sperm competition might have an impact on the functions of SPP120 (but see below).

Contrastingly, species with non-oral fertilization, SPP120-dependent sperm motility regulation, and SPP120 glycosylation and expression did not form viscous milt and the immobilization/aggregation of spermatozoa did not occur. Substrate fertilization species release spermatozoa onto eggs after egg release; hence, spermatozoa immobilization is not useful for fertilization success (Fig. 2c; Additional file 4: Movie S4). Similar to substrate fertilization, spermatozoa immobilization did not contribute to the success of stocked-spermatozoa fertilization because diffused sperm was used for fertilization (Fig. 2b; Additional file 3: Movie S3) [15]. In species with non-oral fertilization, SPP120-dependent features seem unlikely to contribute to fertilization success and, because the viscosity of their milt was lower than that of species with oral fertilization (Fig. 6a; Additional file 11: Figure S4), and SPP120 glycosylation, which leads to viscous milt, was rarely detected (Fig. 5b; Fig. 6b; Additional file 12: Table S1). However, functional modifications in SPP120 also arose in species with non-oral fertilization. These modifications were not related to immobilization or aggregation of spermatozoa (Fig. 8a). Site-specific positive selection in *Cya. furcifer,* a species with stocked-spermatozoa fertilization, and in *Pa. brieni,* a species with substrate fertilization weakened the immobilization/aggregation functions of SPP120 in *Or. mossambicus,* a species with oral fertilization (Fig. 8a, b; Additional file 26: Table S8), indicating that fertilization types influence many aspects of SPP120 evolution.

The phylogenetic tree, showing that a gene duplication event may have occurred in the basal lineage of Tanganyikan cichlids (Fig. 8b; Additional file 23: Figure S9), also demonstrated the complex evolution of SPP120. Differences in the topology of SPP120 (Fig. 8b; Additional file 23: Figure S9) and mitochondrial trees (Fig. 3a) may be due to incomplete lineage-sorting, introgression, or hybridization among these cichlids [37]. A concerted evolution seems likely for each species, with many gene copies arising via species homogenization (Additional file 23: Figure S9). In abalones, homogenizing repeats of one region of the fertilization-related protein vitelline envelope receptor of lysin (VERL) occurred within a population (e.g., [38–40]). In the case of VERL, gene conversion arose during speciation, and alterations in VERL repeats affected its function of species recognition at the time of fertilization [40]. Similarly, SPP120 emerged as a result of gene conversion [6]; thus, the concerted evolution of SPP120 within a population is possible via an increase in the number of SPP120 copies. Although the rate of molecular evolution in most of the SPP120 copies was greater than one (Fig. 8b; Additional file 24: Table S6), the rate of molecular evolution of the most repeated area in VERL was relaxed [38, 41, 42].

The codon site model suggested that positive selection occurred in all SPP120 groups, but the sites undergoing positive selection differed among groups (Fig. 8b; Additional file 24: Table S6). Additionally, SPP120 functions differed among species (Fig. 8a). Loss of SPP120 function was observed in the species with non-oral fertilization, while SPP120 caused spermatozoa immobilization in the species with oral fertilization (Fig. 8a, b); mutations in positively selected sites caused functional modifications (Fig. 7a; Fig. 8a). Overall, the functions of SPP120 changed according to fertilization type. Although the evolutionary history of SPP120 was not examined in the present study, the functional differences among the sequences of reconstructed SPP120 at the deep lineage nodes, where substrate fertilization dominated, might have been affected by the transition from substrate fertilization to another fertilization mechanism.

The implications of sperm competition are reasonable, but the evolutionary correlation with the functions of SPP120 have not been elucidated. Many studies have shown that sperm competition affects the evolution of fertilization in relation to seminal plasma proteins [2, 4]. These studies show that mating systems, such as polyandry, lead to the risk of multiple mating, and thus, seminal plasma proteins that affect sperm availability and longevity, might have evolved. In other words, seminal plasma proteins could have evolved if they affected fertilization rates. The predictions of sperm competition on SPP120 evolution are rational because sneaking male could send milt to female mouth. Sperm availability for fertilization is associated with SPP120 when sperm from two or more males are present in the female mouth. However, strong correlations between glycosylation of SPP120 and *SPP120* expression leading to diversification of SPP120 functions have not been detected. It is predicted that sperm competition in the oral cavity rarely occurs because sneaking behavior may not occur frequently. Further studies are required to evaluate the influence of sperm competition on SPP120 evolution.

In summary, the fertilization-related SPP120 may have evolved to increase the success of a specific spawning behavior (i.e., oral fertilization) in cichlids, as this protein arose via gene conversion [6] at approximately the same time as oral fertilization behavior. SPP120 has multiple functions like increasing milt viscosity via its glycosylation and regulation of sperm motility (immobilization/aggregation), which contribute to the success of oral fertilization. While our results suggest that fertilization types have driven the evolution of SPP120 function, it will be interesting to examine whether other forces, such as sperm competition, have also played a role.

## Methods

### Animals

In this study, we analyzed fifteen mouth brooding species, which exhibit fertilization in the oral cavity, nine bower-building species, which exhibit stocked-spermatozoa fertilization, nine mouth-brooding species with substrate fertilization, eleven substrate-brooding species with substrate fertilization, and one mouth brooding species with an unknown fertilization type (*N* = 3 to 7 males for the analysis; Additional file 12: Table S1; Additional file 29). All fish were captured using a gill net (1.2 m × 7 m, 2.0 mm^2^) or fishing hook at the southern region of Lake Tanganyika, Republic of Zambia, and anesthetized with FA100. Fish were then dissected, and their testes were collected. The testes were store in RNAlater solution (Ambion, Austin, TX, USA) for transportation from East Africa to Japan. Five volumes of RNAlater were used to store one volume of the testes. During the transportation from Zambia to Japan (approximately 3 days), testes in RNAlater solution were stored in a container with cold storage materials, or at room temperature (about 20 to 30 °C). Total RNA from each testis was successfully extracted. Protein analysis was successfully performed for testes stored in both the aforementioned conditions. Three individuals of each species were used for the analysis of SPP120 glycosylation and SPP120 expression. Animal experiments were performed as per the regulations of the Guide for Care and Use of Laboratory Animals, and all experimental protocols were approved by the Committee of Laboratory Animal Experimentation at the University of the Ryukyus.

## Observation of spawning behaviors

We observed spawning behaviors at Nkumbla (Mbita) island (8°45’S, 31°6′E), Cape Kaku (8°36’S, 30°50′E), Mutondwe island (8°43’S, 31°7′E), and Kasenga Point (8°42’S, 31°8′E) according to Morita et al. [15]. Fish spawning behavior using scuba diving equipment was carried out for 7 species of mouth brooders through direct observation or by using a digital camera in an underwater housing (FinePix F11, Fujifilm, Tokyo, or PowerShot G12, Cannon, Tokyo).

### Reconstruction of ancestral states of fertilization types and their transitions during speciation

To investigate the transitions among fertilization types, the ancestral state had to be determined. Fertilization types were categorized into oral fertilization (Fig. 2a; oral fertilization), stocked-spermatozoa fertilization in bower-building species (Fig. 2b; stocked-spermatozoa fertilization), and substrate fertilization in mouth and substrate brooders (Fig. 2c; substrate fertilization). Ancestral state reconstruction was based on tree files constructed using the mitochondrial genes *ND2* (NADH-dehydrogenase subunit 2) and *Cytb* (Cytochrome *b*), or a concatenated gene dataset [43] in BayesPhylogenies (505,000 iterations; burn-in 5000) [44]. Tree files and multi-state trait files, which included fertilization types and species names, were analyzed in BayesTraits version 2.0 [29, 30]. Nodes were categorized and the probabilities of three fertilization types were calculated using the AddMRCA command by applying maximum likelihood estimation. Nodes with high probability for each fertilization behavior were statistically examined to determine which fertilization type was preferred over others using the Fossil Command, after categorizing fertilization types as binary traits, setting the tested type (e.g., substrate fertilization) as “1” and all others as “0.” Next, the likelihood ratios of target nodes were calculated using the nodes set as “1” or “0”, and then a chi-square test was performed to determine whether the likelihood of state “1” was dominant or not using: 2 × (log*H*_dominant(1)_ - log*H*_not dominant(0)_).

To examine transitions of fertilization types among species, multi-state analysis were also conducted in Reverse Jump MCMC analysis [45] setting a hyper prior exponential distribution from 0 to 100. Ancestral reconstructions suggested that the transitions from oral to substrate or stocked-spermatozoa fertilization, or from stocked-spermatozoa to substrate fertilization might not have occurred and therefore, in the next step, the co-efficient of these transitions were restricted to “0” (Q_oral to substrate_ = Q_stocked-spermatozoa to substrate_ = Q_oral to stocked-sperm_ = 0). The harmonic means of these two analyses were then used to calculate the Bayes Factor (BF): 2 × (Harmonic mean _(complex model: non-restricted analysis)_ - Harmonic mean _(simple model: restricted analysis)_). The values of BF were categorized as follows: BF < 2 indicated weak evidence, 2 < BF < 5 indicated positive evidence, 5 < BF < 10 indicated strong evidence, and BF > 10 indicated very strong evidence.

### Fertilization trials

The contribution of seminal plasma proteins to oral fertilization success was investigated using *Or. mossambicus*. Before fertilization trials, milt and eggs were removed by pressing the abdomens of males and females, respectively, and collected by using a 200-μl pipette. Fertilization trials were conducted within 10 min of the collections.

To assess the effect of seminal plasma proteins, ASP was added to milt. The milt of *Oreochromis* spp. was highly viscous [10], which was also the case in *Or. mossambicus*. Milt viscosity was decreased by mixing 10 μl *Or. mossambicus* of milt (5 × 10^8^ ~ 2 × 10^9^ spermatozoa/ml with more than 80% motility) with 40 μl of ASP (143 mM NaCl, 50.7 mM KCl, 0.18 mM MgSO_4,_ 10 mM HEPES-NaOH, pH 8.0) in a 1.5 ml vial. This suspension was then transferred to a mesh cup (200-μm plankton net; Additional file 9: Figure S3a) containing 20–40 eggs. In a similar trial, 10 μl of intact viscous milt (without mixing with ASP) was transferred to an identical mesh cup, and fertilization was performed using the same procedure (Additional file 9: Figure S3a). A control experiment was carried out without using mesh cup. Fertilization success was assessed by inspecting egg division after 1 h of fertilization and confirmed after 8–16 h based on egg development.

### Detection of glycosylated proteins in the seminal plasma or testes

The glycoproteins present in the seminal plasma of *Or. mossambicus* were examined to determine its increased milt viscosity. Seminal plasma was isolated as the supernatant resulting from milt centrifugation at 12,000×*g* for 10 min at 4 °C. Five volumes of lysis buffer (8 M urea, 2 M thiourea, 100 mM dithiothreitol (DTT), 2% (*w*/*v*) CHAPS) was then added to the seminal plasma. Glycoproteins in the testes of *P. fasciolatus* and *Cya. furcifer* were also analyzed by 2D polyacrylamide gel electrophoresis (PAGE). As the testes were preserved in RNAlater solution, stored testis was eluted with lysis buffer and the supernatant was used for the following analyses.

To isolate proteins according to their isoelectric points, 300 μg of each protein was applied to a 10-cm immobilized pH gradient (IPG) gel (pH 3–10) (GE Healthcare, Buckinghamshire, England) and isoelectric focusing was carried out according to Nomura et al. [46]. For 2nd dimension, sodium dodecyl sulfate polyacrylamide gel electrophoresis (SDS-PAGE) with a 3.5% stacking gel and 10% resolving gel was used. Staining of seminal plasma proteins including SPP120 was done using Coomassie brilliant blue, whereas unstained gels were subjected to lectin blotting to detect glycoproteins or SPP120. To detect SPP120 in the forty-four species of Tanganyikan cichlids, proteins were eluted in a solution of 150 mM NaCl and 20 mM Tris-HCl (pH 7.4), and subjected to 1D SDS-PAGE.

To detect glycoproteins, lectin blotting was carried out. Seminal plasma proteins and testes proteins were separated by 2D or 1D SDS-PAGE and transferred to polyvinylidene fluoride (PVDF) membranes. Mochida et al. [8, 9] showed that glycoproteins in the seminal plasma of *Or. niloticus* were ConA-positive N-glycosylated, and thus, the lectin blot was developed using biothynilated-ConA. To detect SPP120, western blotting was performed with rabbit anti-SPP120 antibody (1:10,000) and horseradish peroxidase (HRP)-conjugated anti-rabbit IgG (1:50,000) diluted in blocking buffer. For biotinylated ConA, streptavidin HRP (1/5000 dilution) in blocking buffer were used. Briefly, before the first antibody reaction and lection binding, the PVDF membranes were blocked overnight at 4 °C with 5% skim milk prepared in TBS containing Tween-20 (TBST). The signal was then detected with SuperSignal West Dura Extended Duration Substrate (Thermo Scientific, Waltham, MA, USA).

### Deglycosylation of seminal plasma proteins

Glycosylated SPP120 were subjected to enzymatic deglycosylation by adding 2 μl of glycopeptidase-F (Takara, Otsu, Japan) to 10 μl of a seminal plasma sample prepared as described earlier (see “Detection of glycosylated proteins in the seminal plasma or testes”), and kept overnight at room temperature (22 to 26 °C). Samples were then analyzed using 7.5% SDS-PAGE and western blot.

### Milt diffusion

To examine differences in milt viscosity in different fertilization type, milt diffusion was evaluated in species with oral fertilization (*P. fasciolatus*, *Or. mossambicus*, and *Op. ventralis*), substrate fertilization (*V. moorii* and *L. elongatus*), and stocked-sperm fertilization (*Cya. furcifer*, and *Op. nasuta*). Ten microliters of lake water were placed on each cover slip and 2 μl of milt from each of the seven species were added on the respective cover slips. Diffusion of milt was monitored using a digital GX-10 camera (Cannon, Tokyo, Japan). Sperm movement in the milt was observed under a microscope (Daiko Science Co. Ltd., Tokyo, Japan) after dropping it onto lake water.

To examine the glycopeptidase-F treated milt of *Or. mossambicus*, a 10 μl milt sample was stained with 1 μl of bromophenol blue (1% *W*/*V*), dropped on the hypotonic solution (50 mM NaCl, 10 mM HEPES-NaOH, pH 8.0) on a cover slip, and monitored as described above.

Diffusion rates were calculated based on movie files (MOV files) edited in Adobe Premiere Pro CS5.5 (Adobe, Jan Jose, CA, USA) and observed with ImageJ 1.44 (NIH, Bethesda, MD, USA) at an 8-bit grey scale. Milt areas before-dilution and 3 s after dilution were measured in pixels after selecting the area using the freehand tool.

### Transition in SPP120 glycosylation and its evolutionary correlation with oral fertilization

Glycosylation of SPP120 is likely related to milt viscosity, which observed in most of the species with oral fertilization. Transition from non-glycosylated to glycosylated SPP120 appears to have arisen concomitantly with oral fertilization. BayesTraits version 2.0 was used to test the evolutionary correlation between the binary traits defining each fertilization type and SPP120 glycosylation. First, two glycosylation states of SPP120 (non-glycosylated and glycosylated) were defined based on the SPP120 western blotting results. When the large band was not detected, SPP120 was considered to be non-glycosylated. The transition of SPP120 from a non-glycosylated to a glycosylated state was then examined in multi-state analysis carried out using the mitochondrial and nuclear trees.

To analyze the evolutionary correlation between oral fertilization and SPP120 glycosylation, species with oral fertilization were categorized as “1” (i.e., presenting SPP120 glycosylation) and species with other fertilization types were categorized as “0”. Two models (discrete-independent and discrete-dependent), based on the mitochondrial and nuclear trees, were used to test if oral fertilization was correlated to SPP120 glycosylation. The maximum likelihood ratio of these two models was calculated as (2 × (log Likelihood_dependent_ – log Likelihood_independent_)), and a chi-square test was conducted with seven degrees of freedom. Analysis supported the dependent model and, therefore, the “discrete-dependent” model was run at Reverse-jump MCMC [45] with a hyper-prior distribution (gamma = 0–10).

### Phylogenetic relationships based on fertilization types and SPP120 function

The glycosylation of SPP120 and its function might be evolutionarily correlated. To investigate the phylogeny of fertilization types and SPP120 function, a PGLS analysis [47–51] was performed in R (ver 3.10) [45, 50, 51]. This analysis accounts for the expected co-variance of species traits, which is based on the phylogeny and on the model of trait evolution. Phylogenetic trees, based on the combination of *ND2* and *Cytb* data, concatenated cichlid data according to BS Meyer, M Matschiner and W Salzburger [43], or *SPP120* data using RAxML [52] (rapid bootstrap and general time reversible-gamma model), were used as phylogenetic data files, and PGLS analyses were conducted using a lambda estimated according to maximum likelihood. Lambda denotes the mode of evolution: lambda = 0 means that traits evolved independent of phylogeny, while lambda = 1 denotes that traits co-varied in direct proportion to the phylogenetic distance to their nearest common ancestor [53].

### Functional assay of SPP120

SPP120 is involved in spermatozoa immobilization, which does not seem to contribute to the success of fertilization in the species with substrate or stocked-spermatozoa fertilization. In addition, SPPs have three domains, vWD, C8, and ZP3, however, which of these are functionally important is not known. To investigate the functions of SPP120, functional assays were carried out using the expressed proteins.

SPP120 was expressed in pCold I DNA or pCold ProS2 (both from Takara). Total RNA was extracted from freshly collected testes (*Or. mossambicus*) or testes preserved in RNAlater (Tanganyikan cichlids) using the TRIzol reagent (Thermo Fisher, Waltham, MA, USA), and cDNA was synthesized from testicular total RNA using AMV reverse transcriptase (Takara) or SuperScript III (Invitrogen, Carlsbad, CA, USA) using oligo-dT primers. Target sequences for SPP120 constructs were amplified with PrimeStar GLX (Takara) using several primers (Additional file 12: Table S1; Additional file 29). Amplified inserts were treated with *Eco*RI/*Xho*I (37 °C for 7 h) and ligated into vectors with the DNA Ligation Kit-Mighty Mix (Takara) at 4 °C, overnight. Copy number of ligated constructs were amplified by transformation into *E. coli* DH5α (Takara), and plasmids were isolated using NucleoSpin Plasmid QuickPure kit (Macherey-Nagel, Duren, Germany). Amplified constructs were transformed into *E. coli* BL21(DE3) to obtain FullSPP(F0R0.2), NofSPP(F0R2), ZPlcSPP(F0R1), NlcSPP(F0.8R0.2), NVlcSPP(F0.9R0.2), and ZPofSPP(F1.9R0.2) tags. Expressed proteins were purified by using HisTALON buffer set (ClonTech, Mountain View, CA, USA). Eluted proteins (Additional file 29) were re-suspended in 10 volumes of refolding buffer (50 mM Tris-HCl, 1 mM EDTA, 1 M L-arginine, 1 mM glutathione (reduced), and 0.8 mM glutathione (oxidized) pH 8.3) and incubated overnight at 4 °C. These suspensions were then dialyzed against 50 mM Tris–HCl (pH 8.0) for 6 h at 4 °C.

In the spermatozoa immobilization assay, *Or. mossambicus* spermatozoa were suspended in 30 μl of sperm activation solution (50 mM NaCl, 10 mM CaCl_2_, and 10 mM HEPES-NaOH, pH 8.0) on bovine serum albumin-coated glass slides as described previously [54]. Three microliters of expressed SPP120 (FullSPP, NlcSPP, NofSPP, or ProS2) suspensions (50 mM Tris–HCl (pH 8.0), 100 μg/ml protein) were then added to this suspension to evaluate sperm immobilization (final concentration = 10 μg/ml of expressed proteins). To assess the effect of expressed SPP120 on sperm motility/aggregation, spermatozoa movements and flagellar motility were observed under dark field illumination, video recorded (DCR-TRV70; Sony, Tokyo, Japan), and photographed with a CCD camera (63W1N; Mintron Enterprise Co., Ltd., New Taipei City, Taiwan) mounted on the microscope (Nikon OptiPhoto, Tokyo, Japan). In the spermatozoa aggregation assay, we used recombinant SPP120 (FullSPP, NlcSPP, or NofSPP). To observe sperm aggregation effect by recombinant SPP120s, spermatozoa were first suspended into a Ca^2+^-free activation solution containing recombinant SPP120s (50 mM NaCl, 30 μg/ml expressed SPP120, and 10 mM HEPES-NaOH, pH 8.0). In this solution, sperm motility was not fully activated. Three microliters of 100 mM CaCl_2_ solution (final concentration = 10 mM) were then added to activate sperm motility. Activated sperms gradually aggregated with each other in the presence of functional recombinant SPP120.

To examine the interaction of SPP120 with sperm or with other SPP120, cross-linking experiments and far-western analysis were conducted. Cross-linking experiments were performed using 2.5 mM DSS [55] (2 μl) in dimethyl sulfoxide, which was added to 50 μl of SPP120 suspensions (300 μg/ml, final concentration = 100 μM) and maintained at room temperature (22 to 26 °C) for 2 h. The reaction was terminated by adding 5× SDS sample buffer. In the far-western analysis, 10 μg of triton soluble fraction or remnant spermatozoa of *Or. mossambicus* were subjected to SDS-PAGE, using a 10% gel, and separated proteins were transferred to PVDF membrane. Expressed FullSPP120 (100 μg/ml) was used in the analysis of SPP120 interactions with spermatozoa proteins on PVDF membranes (Additional file 29).

### Isolation of SPP120 cDNA clones

To investigate the molecular evolution of SPP120, cDNA cloning was performed for 31 species of Tanganyikan cichlids. Fragments of SPP120 were amplified with ExTaq (Takara) using several primer sets, and their 3′ or 5′ ends were rapidly amplified (Additional file 29). The PCR products were ligated into the pGEM-T Easy vector (Promega, Madison, WI, USA), and transformed into JM109 competent cells (Takara). Plasmids were then extracted, and sequencing was performed by Macrogen Japan (http://www.macrogen-japan.co.jp/). Cycle-sequencing reactions were conducted with ABI BigDye Terminator v3.1 Cycle Sequencing Kits, and AmpliTaq DNA polymerase (FS enzyme; both Applied Biosystems, Foster City, CA, USA), followed by capillary electrophoresis in the ABI 3730xl sequencer (Applied Biosystems, Foster city, CA, USA).

### Semi-quantitative PCR

Semi-quantitative PCR was performed for 44 Tanganyikan cichlid species using *β*-actin as the internal control. Reaction mixtures comprised 10 μl of GoTaq Green Master Mix (5 μl GoTaq, 4 μl nuclease-free water, 0.5 μl 10 mM forward primer, and 0.5 μl 10 mM reverse primer) and 0.5 μl cDNA synthesized with SuperScript III (Invitrogen) from 5 μg of total RNA. PCR was performed as follows: 35 cycles at 92 °C for 30 s, 57 °C for 30 s, and 72 °C for 1 min. Three biological replicates were used (i.e., three individuals from each species were tested). Primer sequences are provided in Additional file 29.

### Rate of molecular sequence evolution in SPP120

Rates of molecular sequence evolution in SPP120 were calculated with CODEML in PAML [56]. The complete open reading frames of functional genes from Lake Tanganyika cichlids were used in the analyses, which were carried out using only the functional copies of genes; thus, all pseudogenes from the dataset as well as from the phylogenetic tree were excluded. Splicing variants were also excluded to prevent false positive results. Four clades were formed in the phylogenetic tree of SPP120s (Fig. 7c; Additional file 27: Figure S10), and the putative function of SPP120 in species fertilization was likely to differ among clades. Thus, the codon site model (Model 8 vs. 8a) was used to examine differences among groups regarding selected sites via Bayes empirical Bayes analysis [57, 58].

In addition, several species showed different fertilization types within clades. Thus, to confirm an accelerated molecular sequence evolution in these species (Fig. 7c; Additional file 27: Figure S10), branch-site analysis were conducted to detect selection on specific branches [58, 59]. Branches were assigned to fertilization types and set as foreground when selection was thought to influence the fertilization type being examined. In the null model, the foreground was constrained to be “1.” If the maximum likelihood model included a category of sites with non-synonymous/synonymous mutations (dN/dS) > 1, positive selection likely acted on those sites along that specific lineage. The results of this analysis were compared to those using the codon site model to determine if the sites under positive selection differed according to fertilization type. To confirm the results of branch site analysis regarding changes in fertilization type, tests inverting the foreground and background branches were performed.

## Additional files

Additional file 1: **Movies S1.** Spawning behavior of the oral fertilization cichlid *Oreochromis mossambicus*. (MOV 1020 kb)

Additional file 2: **Movies S2.** Spawning behavior of the oral fertilization cichlid *Ophtalmotilapia ventralis*. (MOV 1450 kb)

Additional file 3: **Movie S3.** Spawning behavior of the stocked-spermatozoa fertilization cichlid *Cyathopharynx furcifer*. (MOV 1270 kb)

Additional file 4: **Movie S4.** Spawning behavior of the substrate fertilization species *Boulengerochromis microlepis*. (MOV 8150 kb)

Additional file 5: **Figure S1.** Distinct fertilization types in Lake Tanganyikan cichlids and ancestral reconstruction of fertilization types. (PDF 562 kb)

Additional file 6: **Table S4.** Results from phylogenetic generalized least squares (PGLS) analyses of semen diffusion and Glycosylation of SPP120. (XLSX 8 kb)

Additional file 7: **Table S2.** Likelihood ratio test for dominance of fertilization types in nodes. (XLSX 8 kb)

Additional file 8: **Figure S2.** Evolutionary transition of fertilization types by restriction of BayesTraits. (PDF 685 kb)

Additional file 9: **Figure S3.** Diffusion of semen in several species. (PDF 1711 kb)

Additional file 10: **Table S3.** Results from phylogenetic generalized least squares (PGLS) analyses of semen diffusion and fertilization types. (XLSX 8 kb)

Additional file 11: **Figure S4.** Glycosylated proteins, SPP120 positive spots, and CBB stating of testes of the oral fertilization species *Petrochromis fasciolatus*, and the stpcked-spermatozoa fertilization species *Cyathopharynx furcifer*. (PDF 3621 kb)

Additional file 12: **Table S1.** Species, fertilization places and manners, contribution of SPP120 on fertilization, brooding type, Tribe, Mating system, and collection sites. (XLSX 18 kb)

Additional file 13: **Figure S5.** Evolutionary transition of oral fertilization and glycosylation of SPP120. (PDF 770 kb)

Additional file 14: **Movie S5.** Effect of full length SPP120 (FullSPP) on sperm motility. (MOV 2590 kb)

Additional file 15: **Movie S6.** Sperm aggregation in the presence of full length SPP120 (FullSPP). (MOV 403 kb)

Additional file 16: **Movie S7.** Effect of N-terminal of SPP120 (NofSPP) on sperm motility. (MOV 6400 kb)

Additional file 17: **Movie S8.** Effect of SPP120 lacking N-terminal (NlcSPP) on sperm motility. (MOV 629 kb)

Additional file 18: **Movie S9.** Effect of Tris-buffer on sperm motility as a control. (MOV 5430 kb)

Additional file 19: **Figure S6.** DSS cross-linking of ProS2 tag. (PDF 837 kb)

Additional file 20: **Figure S7.** Far-Western analyses of SPP120 on the Triton-soluble fraction (Tx) or remnant fraction of sperm (Ppt) with or without recombinant full-length SPP120 (FullSPP). (PDF 1344 kb)

Additional file 21: **Figure S8.** Immobilization/aggregation effect of SPP120 from various species. (PDF 421 kb)

Additional file 22: **Table S5.** Results from phylogenetic generalized least squares (PGLS) analyses of sperm immobilization and N-terminal region of fertilization types. (XLSX 10 kb)

Additional file 23: **Figure S9.** Phylogenetic tree, motif structure of SPP120s and their molecular sequence evolution. (EPS 3039 kb)

Additional file 24: **Table S6.** Number of species, copy number of *SPP120*, copy number of pseudo-genes, and ratio of pseudo-genes. (XLSX 9 kb)

Additional file 25: **Table S7.** Codon site model and Bayes Empirical Bayes (BEB) analyses in each group. (DOC 25 kb)

Additional file 26: **Table S8.** Test for accelerated evolution in different fertilization manners in the groups. (DOCX 12 kb)

Additional file 27: **Figure S10. ***SPP120* expressions in the testes. (PDF 2532 kb)

Additional file 28: **Table S9.** Results from phylogenetic generalized least squares (PGLS) analyses of *SPP* expression and fertilization types. (DOCX 12 kb)

Additional file 29: Supplementary information. (DOC 58 kb)
