# Supplementary material for: Variations in the breeding behavior of cichlids and the evolution of the multi-functional seminal plasma protein, seminal plasma glycoprotein 120
Source: BMC Evol Biol. 2018 Dec 20;18:197. doi: 10.1186/s12862-018-1292-0 (PMC6302530; doi:10.1186/s12862-018-1292-0)
Supplement: Supplementary file 5 — Figure S1. Distinct fertilization types in Lake Tanganyikan cichlids and ancestral reconstruction of fertilization types. (PDF 562 kb) [file 12862_2018_1292_MOESM5_ESM.pdf]

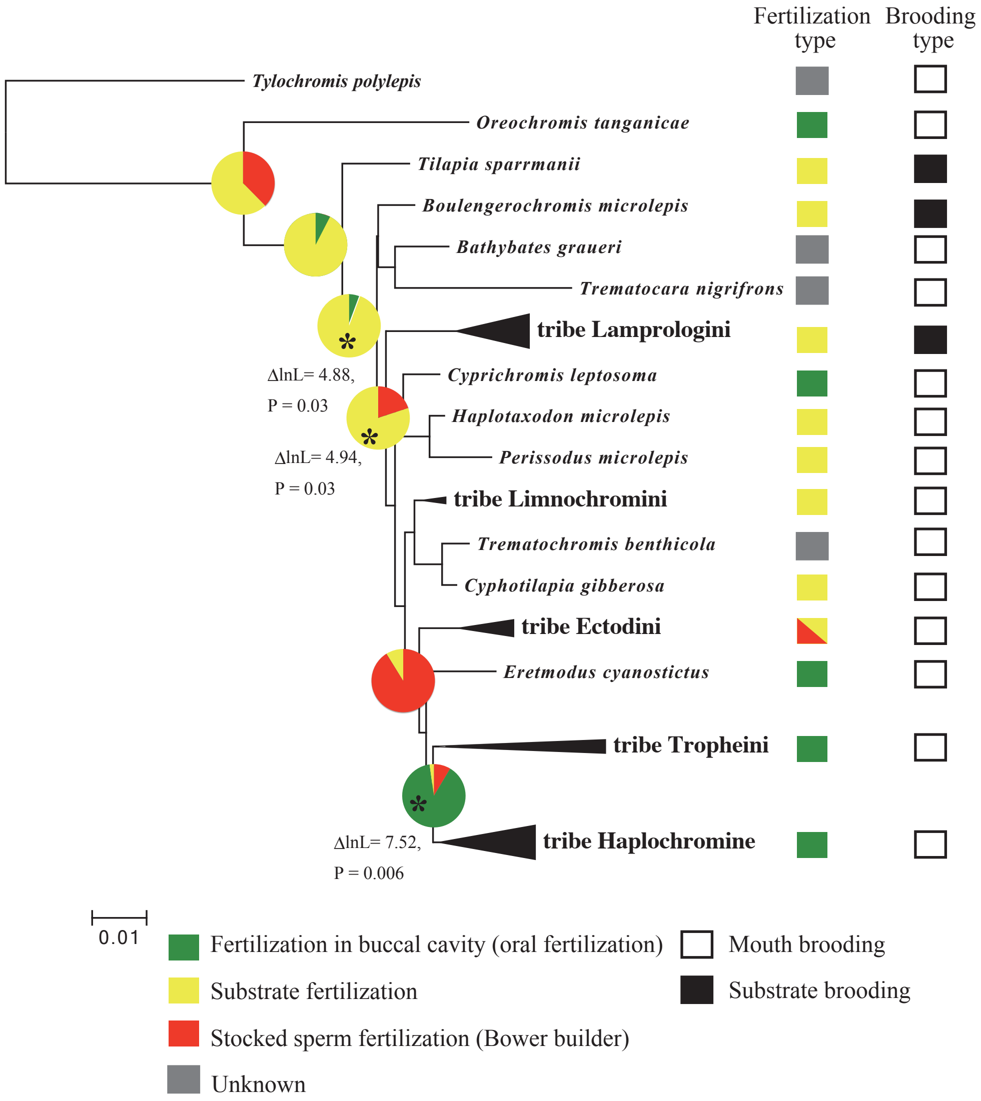

Figure S1 Distinct fertilization types in Lake Tanganyikan cichlids and ancestral reconstruction of fertilization types Fertilization types were categorized into three types: 1) fertilization in the buccal cavity (oral fertilization) in mouth brooders, 2) stocked-sperm fertilization in bower-building mouth brooders, and 3) substrate fertilization in mouth brooders or substrate brooders. Maximum likelihood of the phylogenetic tree based on 45 nuclear markers [43]. Pie charts show the probability of three fertilization types in nodes, and asterisks indicate statistically significant types.
