# Supplementary material for: Variations in the breeding behavior of cichlids and the evolution of the multi-functional seminal plasma protein, seminal plasma glycoprotein 120
Source: BMC Evol Biol. 2018 Dec 20;18:197. doi: 10.1186/s12862-018-1292-0 (PMC6302530; doi:10.1186/s12862-018-1292-0)
Supplement: Supplementary file 8 — Figure S2. Evolutionary transition of fertilization types by restriction of BayesTraits. (PDF 685 kb) [file 12862_2018_1292_MOESM8_ESM.pdf]

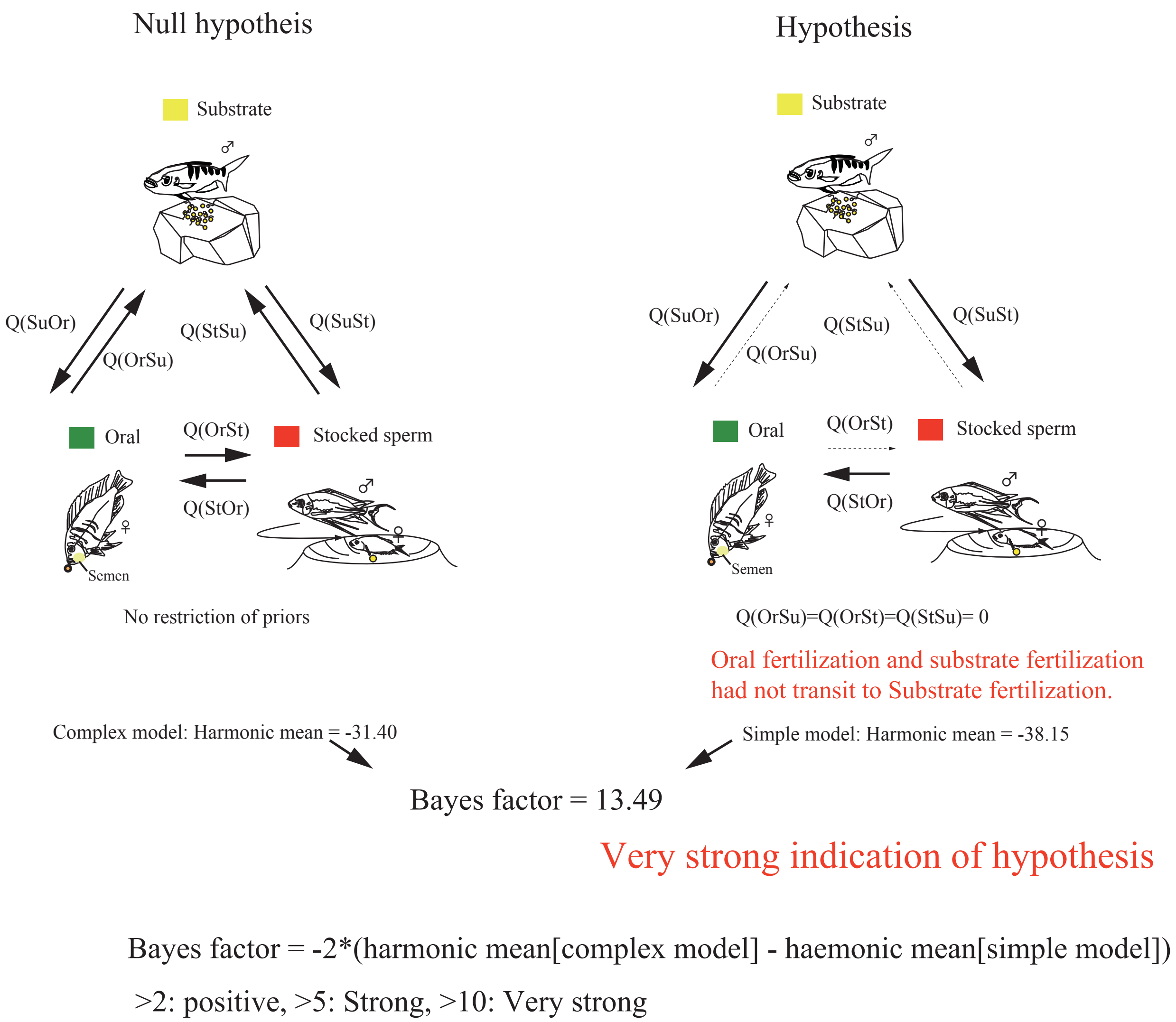

Figure S2 Evolutionary transition of fertilization types by restirction of BayesTraits
