# Supplementary material for: Variations in the breeding behavior of cichlids and the evolution of the multi-functional seminal plasma protein, seminal plasma glycoprotein 120
Source: BMC Evol Biol. 2018 Dec 20;18:197. doi: 10.1186/s12862-018-1292-0 (PMC6302530; doi:10.1186/s12862-018-1292-0)
Supplement: Supplementary file 9 — Figure S3. Diffusion of semen in several species. (PDF 1711 kb) [file 12862_2018_1292_MOESM9_ESM.pdf]

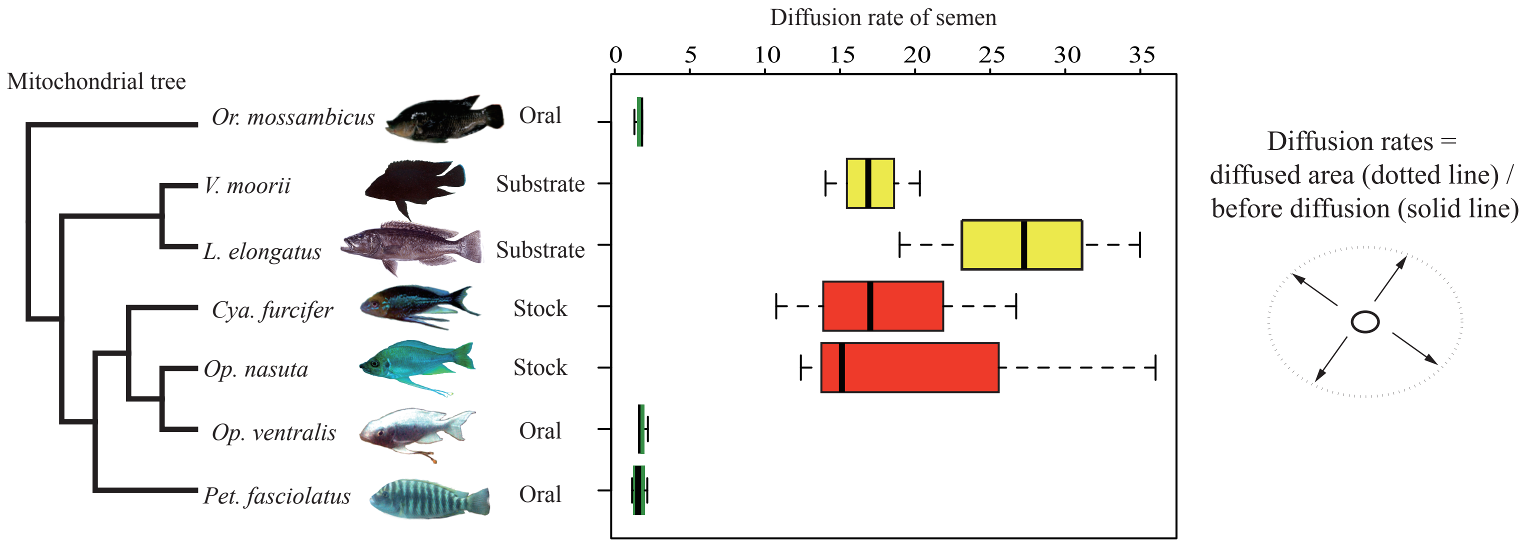

Figure S3 Diffusion of semen in several species

Diffusion rates of semen in terms of fertilization type. Oral fertilization type is following species; *Or. mossambicus* (N=3), *Pet. fasciolatus* (N=4), and *Op. ventralis* (N=4), Bower-building species (stocked-sperm fertilization) are *Cy. furcifer* (N=3) and *Op. nasuta* (N=3), substrate fertilization species are *V. moorii* (N=3), and *L. elongatus* (N=3)
