# Supplementary material for: Variations in the breeding behavior of cichlids and the evolution of the multi-functional seminal plasma protein, seminal plasma glycoprotein 120
Source: BMC Evol Biol. 2018 Dec 20;18:197. doi: 10.1186/s12862-018-1292-0 (PMC6302530; doi:10.1186/s12862-018-1292-0)
Supplement: Supplementary file 11 — Figure S4. Glycosylated proteins, SPP120 positive spots, and CBB stating of testes of the oral fertilization species Petrochromis fasciolatus, and the stpcked-spermatozoa fertilization species Cyathopharynx furcifer. (PDF 3621 kb) [file 12862_2018_1292_MOESM11_ESM.pdf]

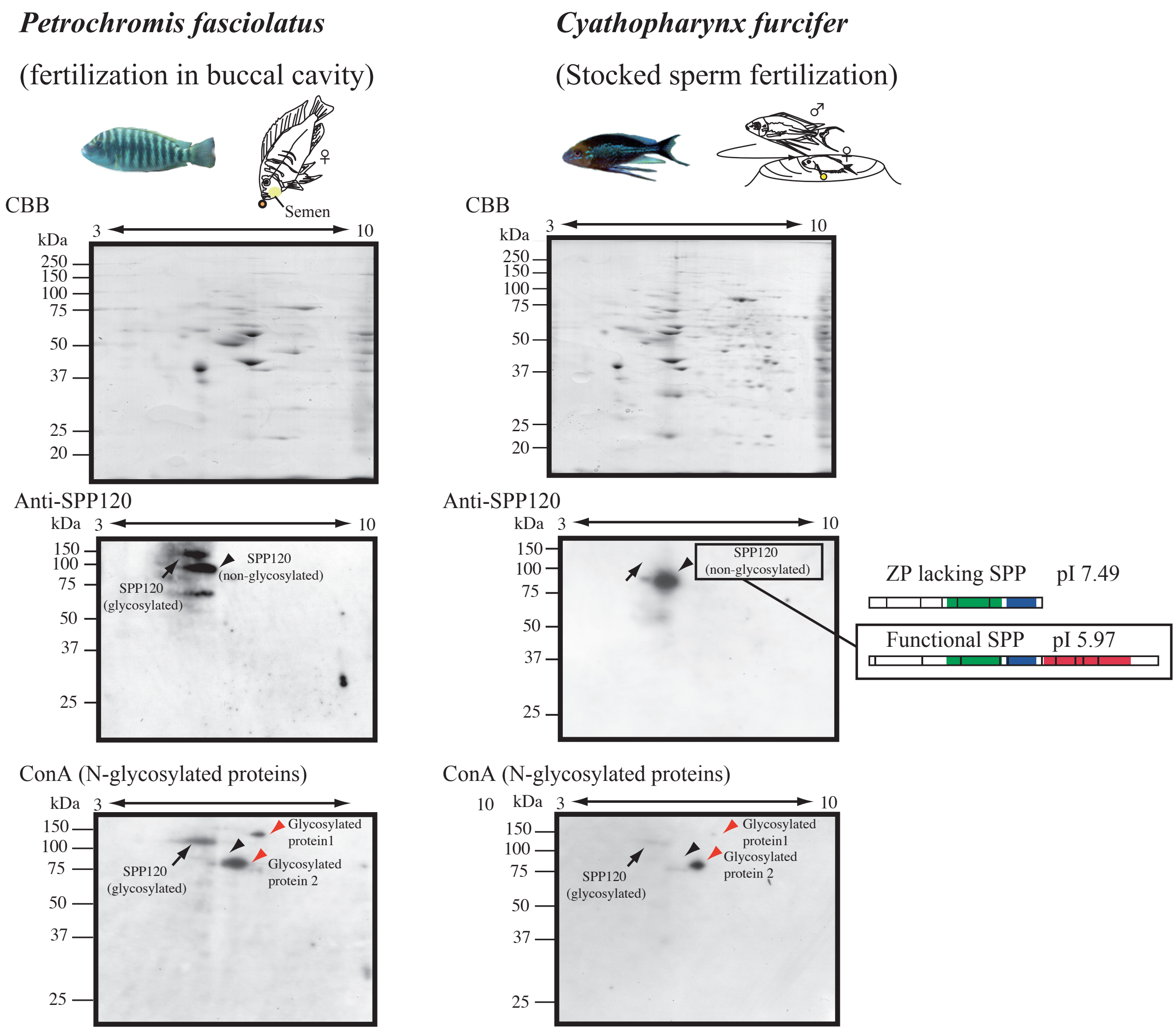

Figure S4. Glycosylated proteins, SPP120 positive spots, and CBB stating of testes of the oral fertilization species *Petrochromis fasciolatus*, and the stpcked-sperm fertilization species *Cyathopharynx furcifer*
