# Supplementary figures and images for: Variations in the breeding behavior of cichlids and the evolution of the multi-functional seminal plasma protein, seminal plasma glycoprotein 120
Source: BMC Evol Biol. 2018 Dec 20;18:197. doi: 10.1186/s12862-018-1292-0 (PMC6302530; doi:10.1186/s12862-018-1292-0)

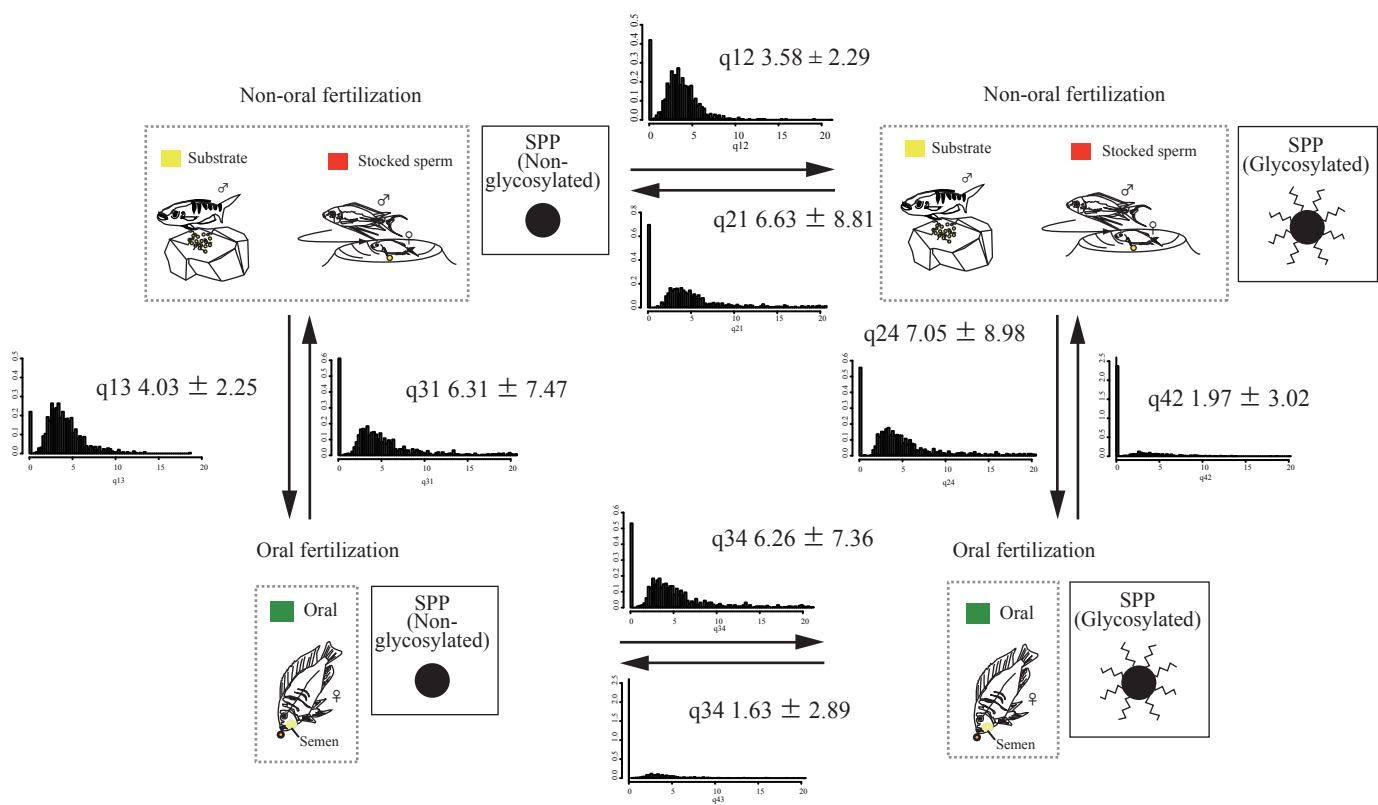

**Figure S5 Evolutionary transition of oral fertilization and glycosylation of SPP120**

Supplement: Supplementary file 13 — Figure S5. Evolutionary transition of oral fertilization and glycosylation of SPP120. (PDF 770 kb) [file 12862_2018_1292_MOESM13_ESM.pdf]

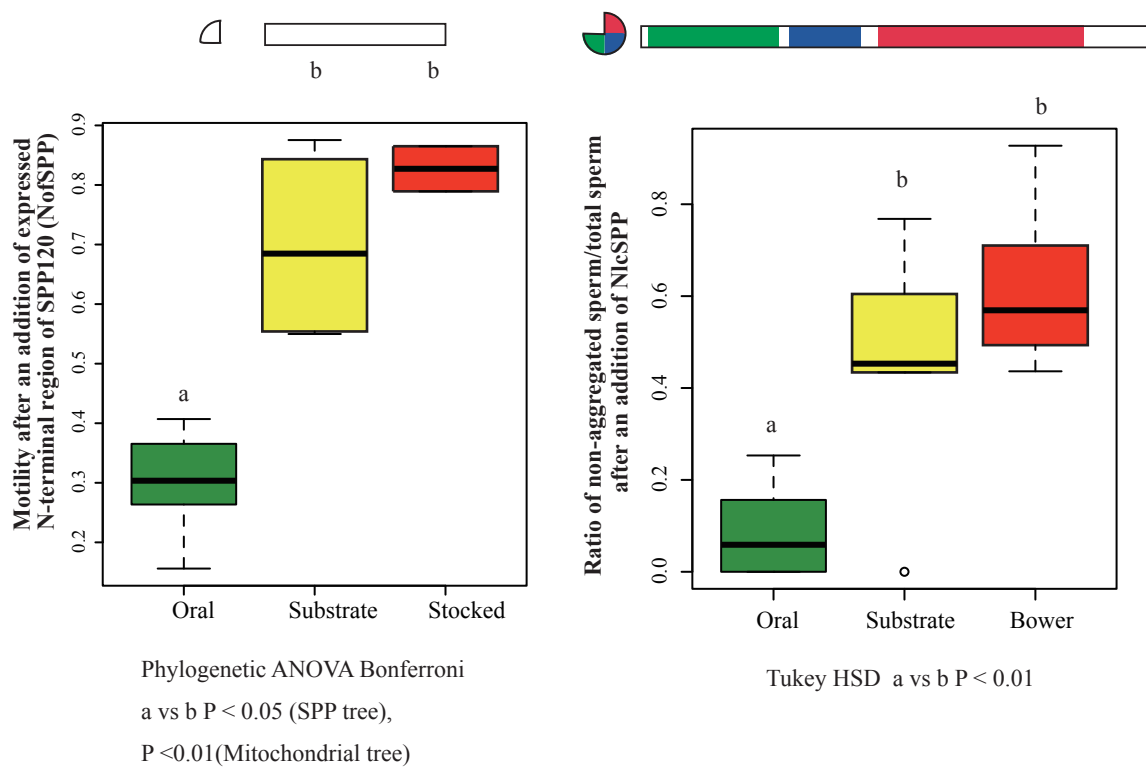

**Figure S8. Immobilization/aggregation effect of SPP120 from various species**

Supplement: Supplementary file 21 — Figure S8. Immobilization/aggregation effect of SPP120 from various species. (PDF 421 kb) [file 12862_2018_1292_MOESM21_ESM.pdf]

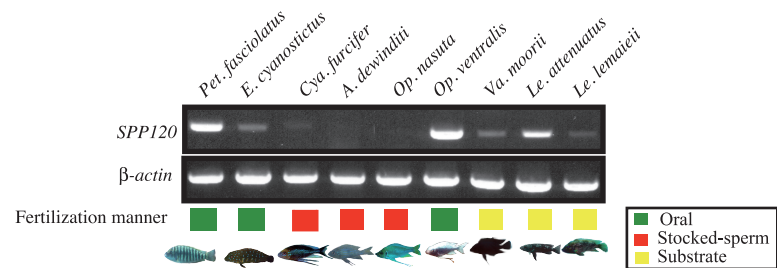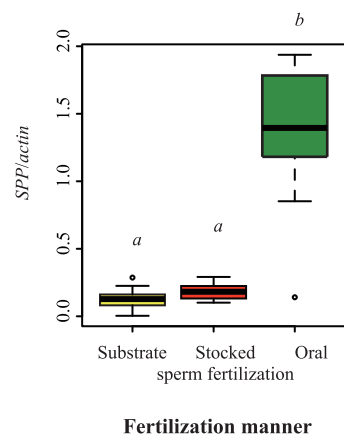

PGLS : supplementary table 8 a vs b :  $P < 0.005$

Supplementary Figure 10. *SPP120* expressions in the testes

Supplement: Supplementary file 27 — Figure S10. SPP120 expressions in the testes. (PDF 2532 kb) [file 12862_2018_1292_MOESM27_ESM.pdf]
