# Supplementary material for: Variations in the breeding behavior of cichlids and the evolution of the multi-functional seminal plasma protein, seminal plasma glycoprotein 120
Source: BMC Evol Biol. 2018 Dec 20;18:197. doi: 10.1186/s12862-018-1292-0 (PMC6302530; doi:10.1186/s12862-018-1292-0)
Supplement: Supplementary file 19 — Figure S6. DSS cross-linking of ProS2 tag. (PDF 837 kb) [file 12862_2018_1292_MOESM19_ESM.pdf]

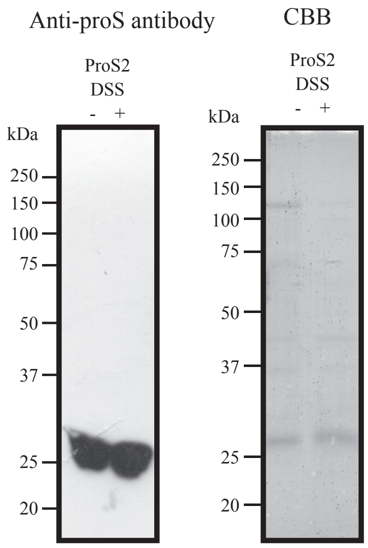


**Figure S6. DSS cross-linking of ProS2 tag.**

Anti-ProS antibody 0.5 ug/ml skim milk TBStween was used for primary antibody and HRP-labelled anti-mouse IgG (20,000 dilution) was used for secondary antibody.
