# Supplementary material for: Variations in the breeding behavior of cichlids and the evolution of the multi-functional seminal plasma protein, seminal plasma glycoprotein 120
Source: BMC Evol Biol. 2018 Dec 20;18:197. doi: 10.1186/s12862-018-1292-0 (PMC6302530; doi:10.1186/s12862-018-1292-0)
Supplement: Supplementary file 20 — Figure S7. Far-Western analyses of SPP120 on the Triton-soluble fraction (Tx) or remnant fraction of sperm (Ppt) with or without recombinant full-length SPP120 (FullSPP). (PDF 1344 kb) [file 12862_2018_1292_MOESM20_ESM.pdf]

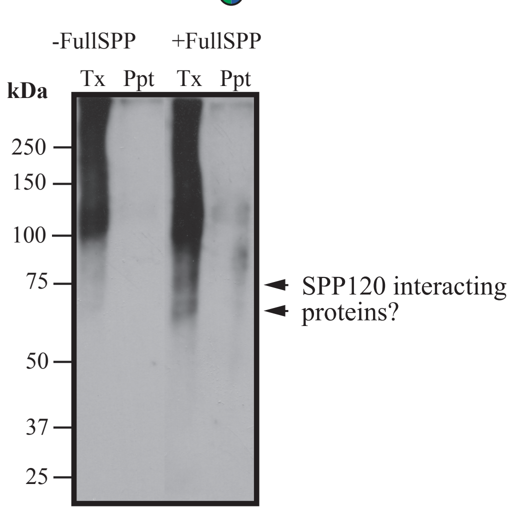

Figure S7. Far-Western analyses of SPP120 on the Triton-soluble fraction (Tx) or remnant fraction of sperm (Ppt) with or without recombinant full-length SPP120 (FullSPP)
