# Supplementary material for: Variations in the breeding behavior of cichlids and the evolution of the multi-functional seminal plasma protein, seminal plasma glycoprotein 120
Source: BMC Evol Biol. 2018 Dec 20;18:197. doi: 10.1186/s12862-018-1292-0 (PMC6302530; doi:10.1186/s12862-018-1292-0)
Supplement: Supplementary file 25 — Table S7. Codon site model and Bayes Empirical Bayes (BEB) analyses in each group. (DOC 25 kb) [file 12862_2018_1292_MOESM25_ESM.doc]

**Table S7 Test for accelerated evolution in different fertilization manners in the groups**

**-lnL -lnL**

**Foreground (Fertilization way) Group Model2Ns2(ω fix) Model2Ns2(ω free)** **ω** **2*Δ*lnL** **p Sites for positive selection**

*Cya. furcifer* and *Au. dewindti* (stock)

Mouth brooder3 4685.1 4678 999 14.2 <0.01* 310 D; vWD domain

*Op. ventralis* (oral) Ectodini -3786.6 3867.6 0.0 >0.05

*Pa. brieni* (substrate) Mouth brooder 2 3724.02 3714.63 136.4 18.8 <0.01* 53 K; N-terminal region
