# Supplementary material for: Variations in the breeding behavior of cichlids and the evolution of the multi-functional seminal plasma protein, seminal plasma glycoprotein 120
Source: BMC Evol Biol. 2018 Dec 20;18:197. doi: 10.1186/s12862-018-1292-0 (PMC6302530; doi:10.1186/s12862-018-1292-0)
Supplement: Supplementary file 29 — Supplementary information. (DOC 58 kb) [file 12862_2018_1292_MOESM29_ESM.doc]

# Additional file 27

# Animals

I used 15 mouth brooding species exhibiting fertilization at buccal cavity (*Petrochromis fasciolatus*, *Petrochromis famula, Petrochromis polyodon, Simochromis pleurspirus, Simochromis diagramma, Interochromis loocki, Ctenochromis horei, Oreochromis mossambicus*, *Oreochromis tanganicae, Cyprichromis leptosoma, Eretmodus cyanostictus*, *Lobochilotes lobiatus*, *Tropheus moorii, Astatotilapia burtoni* and *Ophthalmotilapia ventralis*), 8 bower-building species (*Cyathopharynx furcifer*, *Cyathopharynx foae, Aulonocranus dewindti*, *Benthochromis horii, Cunningtonia longiventralis, Ectodus descampsi*, *Xenotilapia melanogenys*, and *Ophthalmotilapia nasuta*), 9 mouth brooding species with substrate fertilization (*Gnathochromis permaxillaris*, *Greewoodchromis Christy*, *Cyphotilapia gibberosa*, *Paracyprichromis brieni*, *Perrisodus microlepis, Xenotilapia spiloptera,, Xenotilapia flavipinnis*, *Xenotilapia leptura*, and *Xenotilapia boulengeri*), 3 mouth brooding species whose fertilization manners are unknown (*Bathybates grauer)*, and 10 substrate brooding species, three of which are monogamous (*Lepidiolamprologus attenuates, Lepidiolamprologus elongates, Lepidiolamprologus kendali*) and seven of which are polygamous respectively (*Lepidiolamprologus lemairii, Altolamprologus compressiceps, Altolamprologus fascialtus, Termatochromis tempolaris, Neolamprologus modestus, Lamprologus callipterus,* and *Variabilichromis moorii*). With the exception of *O. mossambicus*, which was captured at Nakijin village, Okinawa, Japan, all fish were captured using a gill net at the southern part of Lake Tanganyika, Kasenga Point, Nkumbla Island, Wonzie Point, Mbete Point, or Cape Kachese, Republic of Zambia. Tanganyika. After anesthetizing with FA100, fish were dissected and the testes collected. Testes were stored with RNA later (Ambion) for subsequent transportation from East Africa to Japan.

**Isolation of cDNA Clones of Seminal Plasma Glycoprotein (*SPP 120*)**

RNA was extracted with Trizol (Sigma) according to the manual. cDNA was synthesized with AMV reverse transcriptase (Takara, Otsu, Japan) or Superscript III (Invitrogen, Carlsbad, USA) using oligo-dT primer. Based on full sequences of *SPP 120* *Oreochromis niloticus* (GenBank Accession No. AB073751) and *Astatotilapia burtoni* (EF486262), primers for polymerase chain reaction (PCR) were designed. Forward primers were 5’-ATC TCA GCT GGT TCA TGG TGT-3’ (SPP120-F1), 5’-CTC CRG AAG GTC TTT GTC AGG-3’ (SPP120F(-1)), or 5’-GAT TGG TTC CCT CCA ACA T-3’ ((SPP120 F(-1.5)) and reverse primers were 5’-AAA GAG TTG TTT GCT TGG TAC TTG-3’ (SPP120R1) or 5’-AAA GAG TTG TTT GCT TGG TAC TTG-3’ (SPP120R0). PCR was carried out using cDNA templates. PCR involved 35 or 40 cycles at 92°C for 30 s, 57°C for 30 s, and 72°C for 1 min for SPP120F1-R1, 2 min for SPP120 F(-1)-R1, F(-1.5)-R(1) and F1-R0, or 3 min for SPP120 F(-1)-R0. The PCR products were separated by 1% agarose gel electrophoresis. The PCR product was then ligated and cloned into pGEM-T Easy Vector (Promega, Madison, WI), and sequencing was performed. I sequenced at least eight clones, and although PCR misamplification was removed, many copies showed greater than 1% differences and gaps. Multiple, divergent-transcribed cDNA copies were isolated. *SPP120* is reportedly a multicopy gene [^1^](#_ENREF_1). I isolated many copies from one species and named them (species 01-*v01*) based on numerical order. In addition, if the transcribed copies were 100% identical in coding sequence but gaps were present, these copies were considered to be splice variants from the same loci. Thus, if a greater thajn 1% difference was present among copies, I presumed paralogs named *species* 01 (i.e., *Variabilichromis moorii* 01). If two copies were 100% identical in sequence but had several gaps, they were named *species* 01-*v01* and *species* 01-*v02* (i.e., *V. moorii* 01-*v01*, *V. moorii* 01-*v02*, respectively). Presumably, copies with gaps were splice variants from the same gene locus. The expression of *SPP120* mRNA was assessed using RT-PCR (30 cycles) used as 0.5 µl of cDNA with SPP120 sq-F1 and SPP120 sq-R1 as primers (Supplement Table 1:Table S1), and β-actin was used as a control. For the above PCR analyses, 10 µl of cDNA was synthesized from 400 µg of total RNA with AMV-reverse transcriptase. SPP120 sq-F1 and SPP120 sq-R1 are designed from nucleotide identical region.

# Rapid amplification of cDNA 5’ and 3’ ends

In several cichlids, we conducted rapid amplification of cDNA 5’ and 3’ ends to determine full-length SPP120 cDNA, rapid amplification of cDNA 5’ and 3’ ends (5’- and 3’-RACE) were carried out using the BD Smart RACE cDNA Amplification Kit (BD Niosciences, San Jose, CA) according to manufacture’s protocol. First-cDNA strand cDNA was synthesized using 1 µg of total testes RNA and the primers provided in the kit. The first PCR for 5’- and 3’-RACE was performed with Universal Primer A Mix (UPM) and each gene-specific primer (Table S1). Primer for 3’ RACE was used in common, but primers for 5’ was used for each species. SPP120-5' was used for *C. furcifer* and *C. macrops*, SPP120 5'(ECYA-nest), ECYA SPP120 5'(ECYA) was used for *E. cyanostictus*. SPP120-5'(Pfas) was for *P. fasciolatus*. SPP120 5’(Onus-nest) was for *O. nasuta*. SPP120-5' (Pfas-nest) that was designed from partial sequences by using a 3-step program for touch down PCR. The PCR reactions were as follows: (1) 5 cycles of (1) 5 cycles of 94 °C for 30 s and 72 °C for 3 min; (2) 5 cycles of 94 °C for 30 s, 70 °C for 30 s, and 72 °C for 3 min; and (3) 35 cycles of 94 °C for 30 s, 68 °C for 30 s, and 72 °C for 3 min. Using the 50-fold diluted first PCR products as a template, nested PCR was performed with the Nested Universal Primer A (NUP) and each species-specific nested primers (Table 1) under the following conditions: 25 cycles of 94 °C for 30 s, 68 °C for 30 s, and 72 °C for 3 min. Primer for 3’ RACE (nest) was used in common, but primers for 5’ was used for each species. SPP120-5' nest was used for *C. furcifer*, SPP120 5'(ECYA-nest) was for *E. cyanostictus*, SPP120-5' (Pfas-nest) was for *P. fasciolatus*. SPP120 5’(Onus-nest) was for *O. nasuta*. The final PCR products were ligated and cloned into the pGEM-T easy vector and sequenced. Primers for RACE is shown in Table S1

**Making constructs of SPP120 expression**

PCR was performed with forward- and reverse primers as indicated in Fig. 3a and supporting information (e.g., SPP120_F0 and SPP120_R0.2) from cDNA templates from total RNA. Next, PCR was performed with forward- and reverse primer with restriction enzyme site (e.g., AATA-EcoRI-SPP120_F0 and AATA-XhoI-SPP120_R0.2) from 20 times diluted 1^st^ PCR products. PCR reaction was conducted as follows; 98 ^o^C for 10 sec, 55 ^o^C for 15 sec, 68 ^o^C for 2 min 50 sec (35 cycles). PCR products were cleaned up with Phenol/chloroform and precipitated with sodium acetate and ethanol. The cleaned-up PCR products were digested with EcoRI and XhoI, and cleaned up again with Phenol/chloroform. The digested PCR products were ligated into predigested pCold I or pColdProS2 with DNA ligation kit (Takara) at 16 ^o^C for 3 hours.

**Expression of SPP120**

Colonies were inoculated into 100 ml LB ampicillin and cultured at 37 ^o^C for 7~10 hours until OD600 reached 0.5 to 1. Then, protein expressions were induced with an addition of IPTG (final concentration was 1 mM) and cultured at 15 ^o^C for 24 hour followed by 1 hour incubation at 15 ^o^C. *E. coli* were centrifuged at 10,000g for 5 min at 25 ^o^C and proteins were solubilized with 5 ml extraction solution (8 M urea, 2 M thiourea, and 1%(W/V) CHAPS) and suspensions were dialyzed against 2 M Urea at 4 ^o^C for over night. Dialyzed suspensions were centrifuged at 10,000g for 30 min at 4 ^o^C, and supernatant were applied to TALON gravity column. Wash and equilibration of column and elution of expressed SPP120 were conducted with His TALON buffer set (ClonTech).

**Far-western analyses of SPP120 against sperm proteins**

For far-western analysis, 10 μg of Triton soluble fraction or remnant of sperm in *Or. mossambicus* was subjected to electrophoresis using 10 % gel, and separated proteins were transferred to PVDF membrane. Expressed FullSPP120 (100 μg/ml) was incubated with the PVDF membrane for 1 hour at room temperature followed by 1 hour incubation with blocking solution. The SPP120-treated PVDF membrane was washed with TBS-tween for 5min. Then, to see SPP120 interacted sperm protein on PVDF membrane, SPP120 was detected with anti-ProS2 antibody (0.5 μg/ml) or anti-SPP120 antibody according to western blotting section. Triton soluble fraction was extracted according to [Morita, Takemur^a 2^](#_ENREF_2). About 100 μl of semen were centrifuged at 10,000 g for 5 min at 4 ^o^C and supernatant (seminal plasma) was removed. Then, sperm were re-suspended with 200 μl of artificial seminal plasma (143 mM NaCl, 50.7 mM KCl, 0.18 mM MgSO_4_ and 10 mM HEPES-NaOH pH 8.0) to remove seminal plasma. Sperm suspension was centrifuged at 10,000 g for 5 min at 4 ^o^C. Sperm were washed again with artificial seminal plasma and centrifuged. 200 μl of demembranation solution was added to sperm and incubated on ice for 10 min. Suspensions were centrifuged at 10,000 g for 5 min at 4 ^o^C. Supernatant was removed and lysis solution (8 M Urea, 2 M Thiourea, 100 mM DTT, 2 %(W/V) CHAPS) was added to pellet. Supernatant was used as a Triton soluble fraction and pellet was used as remnant fraction.

**Primers for RACE**

Primers used in 3’ RACE

SPP120-3' 5’- CAG AGC CCT ACA TAA CCG CCT GCA C- 3’

SPP120-3'(nest) 5’- CAG CGA TCA CGA GTT CTG TGG TGA G- 3’

**Primers used in 5’RACE**

SPP120-5' 5’- CAC CGA CCG TCT TCT CAC CAC AGA A- 3’

SPP120-5'(nest) 5’- GCG GTT ATG TAG GGC TCT GGG TCA A- 3’

ECYA SPP120 5'(ECYA) 5’-GCG GTT ATG TAG GGC TCT GGG TCA A- 3’

ECYA SPP120 5'(ECYA-nest) 5’-ACA GCG TTC AGT CAC ACT GGT GCA G- 3’

SPP120-5'(Pfas)　5’- GTC GTC CAG CTG AAC TCT CCC ACC T- 3’

SPP120-5'(Pfas-nest)　5’- CCA GAG GTC TGG AGG GTT GCA GTC T- 3’

SPP120 5'(Onus) 5’-AAG GCC TCA GAG GAT CTT GCA GCA G-3’

ONUS SPP120 5' (Onus-nest) 5’-ACA GCG TTC AGT CAC ACT GGT GCA G-3’

**Primers for semi-q-PCR**

Cichlid_β-actin _F1 5’- CAG GGT GTG ATG GTG GGT A-3’

Cichlid_β-actin _R1 5’- ATG TCA CGC ACG ATT TCC-3’

SPP120_F1 5’- ATCTCAGCTGGTTCATGGTGT- 3’,

SPP120_R1 5’- AAAGAGTTGTTTGCTTGGTACTTG- 3’

**Primers for protein expressions**

SPP120_F0 5’- CTC TGC CCA CTG CTC TTC C-3’

AATA-XhoI-SPP120_F0 5’-AAT ACC TCG AGC TCT GCC CAC TGC-3’

SPP120_F0.8 5’-AAC GAC ATC TGC ATG TTG GA-3’

AATA-XhoI-SPP120_F0.8 5’-AAT ACT CGA GAA CGA CAT CTG CAT-3’

SPP120_F0.9 5’-ACC AGT GTG ACT GAA CGC TGT-3’

AATA-XhoI-SPP120_F0.9 5’-AAT ACT CGA GAC CAG TGT GAC TGA A-3’

SPP120_F1 5’- ATCTCAGCTGGTTCATGGTGT- 3’

AAAA-XhoI-GG-SPP120_F1 5’- AAA ACT TCG AGG GAG GAT CTC AGC T-3’

SPP120_F1.9 5’- AAC AGG GAC ATT GAC CCA CA-3’

AATA-XhoI-SPP120_F1.9 5’-AAT ACTC GAG AAC AGG GAC ATT GAC-3’

SPP120_R0.2 5’-ATA AGT CCA GGA CAC GCT GAT-3’

AATA-EcoRI-SPP120_R0.2 5’-AAt AGA ATT CAT AAG TCC AGG ACA C-3’

SPP120_R1 5’- AAAGAGTTGTTTGCTTGGTACTTG- 3’

TTAT-EcoRI-SPP120_R1 5’-TTA TGA ATT CCA AAG AGT TGT TTG-3’

SPP120_R2 5’-GCA GTC TCA GAA CAG GTG ACA CK-3’

AATA-EcoRI_SPP120_R2 5’-AAT AGA ATT CGC AGT CTC AGA ACA G-3’

SPP_ex_F0 for all 5’-CTGTSCCCACTGCTCTTCC-3’

TATA-XhoI SPP120_ex F0 5’-TATACTCGAGCTGTSCCCACT-3’

SPP_ex_R0 for all 5’-ATAAGTCCAGGACACGCTGATG-3’

AATA-EcoRI SPP120_ex R0 5’-AATAGAATTCATAAGTCCAGGACACGCTG-3’

**References**

1. Gerrard DT, Meyer A. Positive selection and gene conversion in SPP120, a fertilization-related gene, during the east African Cichlid fish radiation. *Mol Biol Evol* **24**, 2286-2297 (2007).

2. Morita M, Takemura A, Nakajima A, Okuno M. Microtubule sliding movement in tilapia sperm flagella axoneme is regulated by Ca2+/calmodulin-dependent protein phosphorylation. *Cell Motil Cytoskeleton* **63**, 459-470 (2006).
